# Neural correlates of sex-related differences in attachment dimensions

**DOI:** 10.3758/s13415-020-00859-5

**Published:** 2021-02-09

**Authors:** Daniela Altavilla, Chiara Ciacchella, Gaia Romana Pellicano, Marco Cecchini, Renata Tambelli, Navkiran Kalsi, Paola Aceto, Carlo Lai

**Affiliations:** 1 Department of Dynamic and Clinical Psychology, and Health Studies, Sapienza University, Via degli Apuli 1, 00185 Rome, Italy; 2 Department of Anaesthesiology and Intensive Care, Catholic University of Sacred Heart, Rome, Italy

**Keywords:** Avoidant attachment dimension, Sex differences, Neural correlates, ERP, sLORETA

## Abstract

The purpose of this study was to investigate sex-related differences in the electrophysiological response to socioemotional stimuli (positive, negative, and ambiguous) depicting couple interactions. The associations between anxiety and avoidance attachment dimensions (measured with the Experiences in Close Relationships–Revised questionnaire) and the strength of cortico-limbic circuit intensity was explored, recorded using a 256-Hydrocel Geodesic Sensor-Net. Event-related potentials (ERPs) and standardized low-resolution electromagnetic tomography (sLORETA) data were analyzed for a total sample of 74 participants. Regression analyses showed that the women presented increased brain intensity compared with that in men, and the avoidance score was positively associated with brain intensity, particularly in response to negative socioemotional stimuli. The interaction sex per avoidance was a significant predictor of intensity in many brain areas, with women displaying significantly more pronounced positive associations between avoidance and brain intensity than men. In conclusion, the findings of the present study showed that women appeared to be more emotionally involved during the socioemotional task. Avoidance was positively associated with intensity of the cingulate and prefrontal regions, and these associations were more pronounced in women than in men. These findings suggested that avoidance seems to represent two different socioemotional strategies, in which women appear to activate an avoidant strategy to modulate increased emotional involvement in relationships, whereas men appear to adopt avoidance with a more intense emotional suppression.

**Supplementary Information:**

The online version contains supplementary material available at 10.3758/s13415-020-00859-5.

Several studies have provided evidence regarding the existence of sex-related differences in socioemotional processing (Collignon et al., 2010; Kret & De Gelder, 2012). According to recent literature, women appear to be considered as more sensitive and focused on emotional aspects of social experiences compared with men (Bianchin & Angrilli, 2012; Guimond, Chatard, Martinot, Crisp, & Redersdorff, 2006). Specifically, women appear to develop a more pronounced tendency to empathize with and understand both the verbal and nonverbal information related to the behaviors of others (Proverbio, Zani, & Adorni, 2008). From an evolutionary perspective, this female propensity could be viewed as a functional adaptation associated with the care of offspring (Hampson, van Anders, & Mullin, 2006). Consistently, the many studies that have investigated sex-related differences in brain activity have indicated the activation of different neural pathways during socioemotional tasks between men and women (Althaus et al., 2014; Bianchin & Angrilli, 2012; Groen Wijers, Tucha, & Althaus, 2013). In particular, some event-related potential (ERP) studies have reported that women prioritize the processing of socially relevant and negative emotional information, showing different P100, N200, and late positive potential (LPP) amplitudes in the occipital, frontocentral, and parietal montages, respectively, compared with those in men (Groen Wijers, Tucha, & Althaus, 2013; Proverbio, Adorni, Zani, & Trestianu, 2009). Moreover, activity in the right amygdala and right prefrontal brain regions in response to pictures depicting characters in negative contexts was only observed in women, suggesting the potential role of these areas in the increased affective response to negative social information in women (Proverbio et al., 2009). Finally, women were shown to present enhanced activity in the cingulate brain areas; this neural correlate could be an expression of women’s propensity to respond empathetically to social stimuli (Proverbio et al., 2008; Sander, Frome, & Scheich, 2007).

In light of these previous studies, the investigation of how sex-related differences affect social and intimate relationships in men and women is of interest (Ratliff & Oishi, 2013). Hazan and Shaver (1987) suggested that adult romantic love represents an attachment process that is affected by infant experiences, theorizing three different forms of attachment styles: secure, anxious/ambivalent, and avoidant. Specifically, secure people feel comfortable with intimacy and maintain autonomy in their relationships, anxious people are extremely preoccupied with relationships and rely on their partners, and avoidant people withdraw from closeness and intimacy in their relationships (Hazan & Shaver, 1987; Main, Kaplan, & Cassidy, 1985).

Many studies have approached this issue from a neurobiological perspective, investigating the brain processes associated with various attachment dimensions (Cecchini, Iannoni, Pandolfo, Aceto, & Lai, 2015; Gillath, Bunge, Shaver, Wendelken, & Mikulincer, 2005; Lai, Altavilla, Ronconi, & Aceto, 2016). In particular, the anxiety dimension of attachment has been positively associated with the activation of emotion-related brain areas (the anterior temporal pole, insula, and anterior cingulate cortex) and inversely correlated with the activation of brain areas involved in emotional regulation processing (orbitofrontal cortex; Gillath et al., 2005).

Previous studies examining the association between the avoidant attachment dimension and cortico-limbic activation (amygdala, insula, anterior cingulate cortex, and prefrontal cortex) have reported contrasting findings (Vrtička, Bomdolfi, Sander, & Vuilleumier, 2012). On the one hand, some studies demonstrated decreased cortico-limbic activity as a function of increased avoidance scores in social exclusion tasks and during the presentation of positive socioemotional stimuli (Dewall et al., 2012; Vrtička, Andersson, Grandjean, Sander, & Vuilleumier, 2008). On the other hand, other studies have shown increased cortico-limbic activation in people with high scores in the avoidant attachment dimension in response to unpleasant socioemotional stimuli (Buchheim, George, Kächele, Erk, & Walter, 2006; Strathearn, Fonagy, Amico, & Montague, 2009; Vrtička & Vuilleumier, 2012). In light of these contrasting findings, whether the avoidant attachment dimension is negatively or positively associated with cortico-limbic activation during socioemotional stimuli processing remains unclear. This divergence in the findings reported by previous studies could be associated with the different socioemotional tasks that were used, which may have resulted in the activation of different neural correlates (e.g., social reward, social exclusion, emotional faces, and crying infants; Lee & Siegle, 2009).

In line with this hypothesis, a very recent metanalytic study suggested that two opposing neurobiological mechanisms may be associated with avoidance (Long, Verbeke, Ein-Dor, & Vrtička, 2020). On the one hand, deactivating strategies may be engaged, which appear to be associated with the relative insensitivity to negative social information, preventing an excessive activation of an “aversion module” (anterior cingulate cortex, insula, hippocampus, amygdala, and anterior temporal pole). On the other hand, avoidance strategies appear to result in the increased sensitivity to negative social information, accompanied by the reduced ability to regulate consequent distress, which results in the increased activation of the aversion module. Simultaneously, positive emotions associated with social contexts also appear to be suppressed. These data suggested that individuals with avoidance attachment present a blunted subjective experience and reduced brain activity in response to positive social information, whereas there is an impaired regulation of strongly negative social information (Long et al., 2020).

Several EEG studies have demonstrated that the N200, P200 and late components appear to be modulated by the attachment style (Krahe et al., 2015; Krahe, Drabek, Paloyelis, & Fotopoulou, 2016; Zayas, Shoda, Mischel, Osterhout, & Takahashi, 2009). Specifically, higher N200 and P200 amplitudes have been associated with increased avoidance (measured with the Experiences in Close Relationships–Revised questionnaire) in response to painful stimuli that occur in the presence of social support (Krahe et al., 2015) or to social rejection/exclusion experiences (White et al., 2012). These social neuroscience data underline how avoidance is associated with the preferential use of suppression as an emotional (self-)regulation strategy during both positive and negative social contexts (Collins & Feeney, 2004; Long et al., 2020), resulting in two possible neurobiological outcomes—either decreased activation or increased sensitivity to social information.

In light of these recent findings, a possible explanation of the contrasting results regarding the relationship between the avoidant attachment dimension and cortico-limbic activation in response to socioemotional stimuli may be ascribable to the adoption of different emotional coping strategies in men and women. In previous studies, positive associations between the avoidance attachment dimension and brain activation were only reported in women (Buchheim et al., 2006; Strathearn et al., 2009; Vrtička & Vuilleumier, 2012), whereas negative associations were either only found only in men or identified in mixed samples (Dewall et al., 2012; Vrtička et al., 2008).

In view of the divergence among these neurobiological studies, the investigation of the associations between attachment dimensions and cortico-limbic activation in men and women is of interest. To the best of our knowledge, few neurobiological studies have focused on this topic.

The primary purpose of this study was to investigate sex-related differences in the electrophysiological responses to socioemotional images of couple interactions. Moreover, the associations between attachment style dimensions and the brain responses in cortico-limbic circuits in both men and women were investigated. The hypotheses were that women would show different amplitudes and latencies for early ERP components in the occipital and temporo-parietal montages, whereas for late ERP components will be observed in the frontal montage, as well as an increased intensity in the limbic, cingulate, and prefrontal cortices in response to socioemotional images compared with the brain intensity observed in men. The association between the avoidant attachment dimension and the intensity of the limbic, cingulate, and prefrontal cortices will be significantly positive and significantly more pronounced in women than in men, particularly in response to images with negative valence.

## Methods

### Participants

Eighty-three right-handed, healthy volunteers participated in the study. The study was approved by the Ethics Committee of the Department of Dynamic and Clinical Psychology, Sapienza University, and all participants signed informed consent to participate in the study. The inclusion criterion was being between 18 and 35 years of age. The exclusion criteria were any history of neurological injury, psychiatric illness, drug abuse, or psychotropic medication.

### Psychological assessment

The Experiences in Close Relationships–Revised scale (ECR-R; Busonera, Martini, Zavattini, & Santona, 2014; Fraley, 2012; Fraley, Waller, & Brennan, 2000; Picardi et al., 2002) was administered to assess two attachment dimensions (anxiety and avoidance). The ECR-R is a 36-item self-report questionnaire that includes 18 items for the anxiety dimension and 18 items for the avoidance dimension (with Cronbach’s α of 0.90 and 0.89, respectively; Busonera et al., 2014). The participants rated each item on a 7-point scale, ranging from 1 (*totally disagree*) to 7 (*totally in agreement*). In the sample used in the present study, the Cronbach’s α values for the anxiety and avoidance dimensions were 0.89 and 0.86, respectively.

### Stimuli

A total of 120 black-and-white pictures were selected. Ninety images depicted a man and a woman engaged in couple interactions, with three different emotional valences, as follows: 30 pictures with couples in intimate interactions (happy facial expressions or direct gaze or physical contact) were chosen for the positive condition; 30 pictures with couples in conflictual interactions (sad or angry facial expressions or averted gaze or physical violence) were chosen for the negative condition; and 30 pictures with couples in neutral interaction (neutral facial expressions or neutral actions or averted gaze) were chosen for the ambiguous condition. Finally, 30 pictures depicting neutral objects were chosen to avoid habituation to the interaction stimuli.

Thirty neutral objects were selected from the International Affective Picture System (IAPS; Lang, Bradley, & Cuthbert, 2008). Ninety pictures of couple interactions were chosen as follows: from the IAPS were selected all the available pictures depicting couple interactions (*n* = 15) and from the internet were selected the remaining pictures (*n* = 75) according to the criteria described above (positive, negative, and ambiguous conditions). All 120 pictures were adjusted for luminance and contrast using the Gnu Image Manipulation Program (GIMP; Version 2.8, Free Software Foundation, Inc.) and Microsoft Office Picture Manager software, setting an identical resolution (640 × 480 pixels) for each image.

A preliminary assessment was performed by 19 volunteers to evaluate the emotional valence (on a Likert scale from 1 = *very negative* to 7 = *very positive*) and the emotional arousal (on a Likert scale from 1 = *no intense* to 7 = *extreme intense*) of each of the 90 couple interaction images.

A repeated-measures analysis of variance (ANOVA), with condition (positive vs. negative vs. ambiguous) as the within-subjects factor, performed on the emotional valence score showed a main effect of the condition, *F*(2, 36) = 283.2, *p* < .001, in which the positive condition reported a higher positive score than the negative (*p* < .001) and ambiguous (*p* < .001) conditions, and the negative condition reported a higher negative score compared with the ambiguous condition (*p* < .001). A repeated-measures ANOVA, with condition (positive vs. negative vs. ambiguous) as the within-subjects factor, performed on the emotional arousal showed a main effect of the condition, *F*(2, 36) = 6.7, *p* = .003, in which the ambiguous condition reported a lower arousal rating than the positive (*p* < .001) and negative (*p* = .039) conditions.

### Experimental procedure

**Fig. 1:**
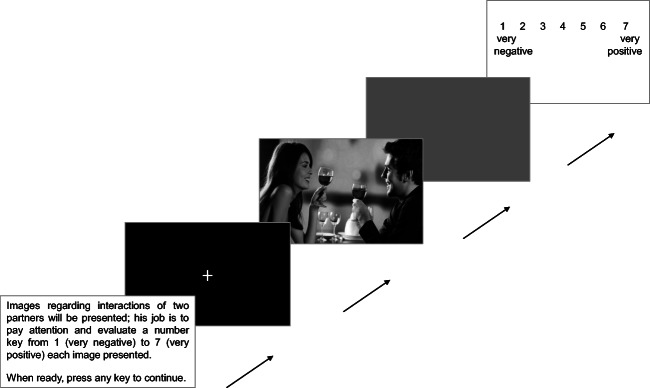
Task procedure. The task started with instructions for the participants. They were instructed to pay attention to the images and evaluate their emotional values. Each trial started with a fixation cross, displayed for 1,000 ms, followed by the picture (positive vs. negative vs. ambiguous vs. neutral), presented for 2,000 ms. Then the participants rated the image on a 7-point scale ranging from 1 (*very negative*) to 7 (*very positive*); the trial ended with an interstimulus interval (ISI) ranging from 300 to 500 ms. A total of 120 trials (30 trials for each condition) were presented in random order

Participants were seated at a viewing distance of 80 cm from a PC monitor (27 cm, 75 Hz, 1,024 × 768). The stimuli were presented using E-Prime (Version 2.0.8.90; Psychology Software Tools, Inc., Pittsburgh, PA, USA). The participants were instructed to pay attention to the images and evaluate their emotional valence. Each trial began with a fixation cross displayed for 1,000 ms, followed by a picture (positive vs. negative vs. ambiguous vs. neutral), which was presented for 2,000 ms. Participants then rated the emotional valence of the visual stimuli on a 7-point scale, ranging from 1 (*very negative*) to 7 (*very positive*). Each trial ended with an interstimulus interval (ISI) with a random duration ranging from 300 to 500 ms. A total of 120 trials (30 trials per condition) were presented in a random order (see Fig. 1). After the visual task, the ECR-R was administered.

### Electroencephalographic (EEG) registration and analysis

Electroencephalographic (EEG) signal was recorded continuously at 250 Hz using Net Station 4.4.2 and a 256-Hydrocel Geodesic Sensor Net, with an impedance below 50 kΩ and referenced to the vertex (Cz).

The acquired data were digitally filtered (30 Hz low-pass) off-line. The EEG data for each subject was segmented into epochs starting 100 ms before the presentation of the stimulus until 700 ms after stimulus onset. The artefact detention was set to 200 μV for bad channels (noisy electrodes), to 150 μV for eye blinks (detected on specific electrodes pairs: left: 37-241/32-241 and right: 18-238/25-238), and to 100 μV for the electrodes detecting eye movements (left: 252 and right: 226; Electrical Geodesic, Inc., Eugene, OR, USA; Bourisly & Shuaib, 2018; Lai et al., 2018; Luan, Yao, & Bai, 2017; Picton et al., 2000; Schreiter, Chmielewski, & Beste, 2018). The segments containing eye blinks, eye movements, or more than 15 bad channels were excluded. Baseline correction −100 ms before stimulus onset was applied.

Through the visual inspection of ERPs components, the following intervals were set: from 80 to 160 ms for the P100, and from 160 to 220 ms for the N200 (early components); from 220 to 300 ms for the P250, from 300 to 500 for the LC1, and from 500 to 700 for the LC2 (late components).

After the EEG signal cleaning from artefacts, as reported by previous studies (Lai et al., 2020; Lai, Pellicano, et al., 2018; Picton et al., 2000; Tanner, Morgan-Short, & Luck, 2015), the following electrode locations were chosen for each montage: occipital (O1, O2), temporo-parietal (left: 65, 75, 84, 86, 97, 107, 108; right: 150, 160, 161, 162, 173, 179, 180), and frontal (left: 22, 23, 24, 28, 30, 47, 49; right: 6, 7, 13, 207, 214, 215, 221).

Data were analyzed on peak amplitudes and latencies for the following: at P100 on the occipital and temporo-parietal montages; at N200 on the temporo-parietal montage; and at P250 on the temporo-parietal and frontal montages. Moreover, at LC1 and LC2, the data were analyzed just on peak amplitudes of the temporo-parietal and frontal montages.

### Source analysis (sLORETA)

The EEG signal was processed through GeoSource 2.0 software (Electrical Geodesic, Inc., Eugene, OR, USA) to identify the locations of the neural generators of the ERP components.

Standardized low-resolution electromagnetic tomography (sLORETA; Pascual-Marqui, 2002), was used to identify source locations based on a probabilistic map of the MNI305 average (Montreal Neurological Institute 305 subjects). Gray matter volume was parcellated into 7-mm voxels, with each voxel serving as a source location with three orthogonal orientation vectors (Cecchini, Aceto, Altavilla, Palumbo, & Lai, 2013; Lai et al., 2016; Lai et al., 2017; Lai et al., 2018; Lai et al., 2020; Lai, Pellicano, et al., 2018; Lancaster et al., 2000; Luciani et al., 2014; Massaro et al., 2018). This parcellation resulted in a total of 2,447 source triplets whose anatomical labels were estimated through the use of a Talairach daemon (Cecchini et al., 2013; Lancaster et al., 2000; Luciani et al., 2014).

In accordance with the hypotheses and based on previous literature (Long et al., 2020; Vrtička & Vuilleumier, 2012) regarding associations between the avoidance dimension and neurobiological responses to socioemotional stimuli, the following regions of interest (ROIs) were chosen for sLORETA analyses: limbic, anterior cingulate cortex, posterior cingulate cortex, and prefrontal cortex. For each ROI, the following Brodmann areas (BAs) in both hemispheres were selected: amygdala, insula, amygdala-hippocampus junction, and hippocampus for the limbic ROI; BA24, BA32, and BA33 for the anterior cingulate cortex ROI; BA23, BA30, and BA31 for the posterior cingulate cortex ROI; and BA09, BA10, BA11, BA46, and BA47 for the prefrontal cortex ROI.

### Statistical analysis

Differences between men and women were examined using t-tests on the number of trials (positive, negative, and ambiguous) without artefacts that were inserted in the final analyses, and on ECR-R scores (anxiety and avoidance).

For behavioral data analyses, the 2 × 3 repeated-measures analyses of covariance (ANCOVAs), with sex (men vs. women) as the between-subjects factor, condition (positive vs. negative vs. ambiguous) as the within-subjects factor, and age and the ECR-R scores (anxiety and avoidance) as covariates, were conducted on the reaction times and the emotional valence assignment of the visual stimuli. Correlation analyses (Pearson’s *r*) were performed between ECR-R scores (anxiety and avoidance) and reaction times, and emotional valence assignment of the visual stimuli. The reaction times and emotional valence assignment scores were subjected to multiple factorial regression analyses with age, sex, ECR-R-anxiety score, ECR-R-avoidance score, and the Sex × ECR-R Score interaction as predictors.

To analyze ERPs data, the 2 × 3 × 2 repeated-measures ANCOVAs, with sex (men vs. women) as the between-subjects factor, condition (positive vs. negative vs. ambiguous) and hemisphere (left vs. right) as the within-subjects factors, and with age and the ECR-R scores (anxiety and avoidance) as covariates were conducted on the amplitude and latency of early and late components on the occipital, temporo-parietal and frontal montages.

For the sLORETA data analyses, a correlation analysis (Pearson’s *r*) was performed between the ECR-R scores (anxiety and avoidance) and the mean intensity of each BA in each ROI in response to each condition for the early and late ERP components (Bonferroni correction was applied, and the *p* value was set at ≤ .0003; 15 BAs × 2 hemispheres × 5 components: 0.05/150 = 0.0003). For each BA in each ROI that showed a significant correlation between the ECR-R-anxiety score, the ECR-R-avoidance score, and the BA mean intensity, a multiple factorial regression analysis was performed, including age, sex, ECR-R-anxiety score, ECR-R-avoidance score, and the Sex × ECR-R Score interaction as predictors.

All statistical analyses were performed with Statistica 10.0 (StatSoft Inc.).

## Results

From among the initial 83 participants, nine were excluded due to the presence of artefacts in the EEG data. The total sample consisted of 74 participants (37 men and 37 women; mean age for men = 24.8 ± 3.9 years; women = 23.8 ± 3.1), *t*(72) = 1.3, *p* = .195.

The *t* tests performed between men and women on the number of positive, negative, and ambiguous trials without artefacts that were included in the final analyses did not show any significant differences (see Table 1).

**Table 1:** The *t* tests performed between men and women on the number of positive, negative, and ambiguous trials without artefacts that were included in the final analyses (*n* = 74)

	Min	Max	Men (*n* = 37) *M* ± *SD*	Women (*n* = 37) *M* ± *SD*	*t*(37)	*p*
Positive trials	6	30	22.7 ± 5.8	20.6 ± 6.3	1.5 1.8 1.1	.142 .069 .271
Negative trials	6	30	21.9 ± 5.7	19.3 ± 6.2		
Ambiguous trials	6	30	21.7 ± 5.7	20.2 ± 5.8		

The mean ECR-R score for the anxiety dimension for men was 3.6 ± 0.8, whereas the mean score for women was 3.5 ± 1.2; the mean ECR-R score for the avoidance dimension for men was 3.0 ± 0.9, whereas the mean score for women was 2.7 ± 0.8. No significant differences were found between men and women for either the anxiety, *t*(72) = 0.5, *p* = .635, or avoidance, *t*(72) = 1.4, *p* = .156, dimensions.

### Behavioral data

A 2 (sex: men vs. women) × 3 (condition: positive vs. negative vs. ambiguous) repeated-measures ANCOVA, with age and ECR-R scores (anxiety and avoidance) as covariates, performed on the reaction times and emotional valence assignment of the visual stimuli showed the following results. For the reaction times, a main effect of the condition was identified, *F*(2, 138) = 3.9, *p* = .023; ƞ_p_^2^ = 0.05, in which the ambiguous condition elicited longer reaction times compared with the reaction times for the positive (*p* < .001) and negative (*p* < .001) conditions, and the negative condition elicited longer reaction times than the positive condition (*p* < .001). For the emotional valence assignment in response to visual stimuli, the ANCOVA showed a main effect of the condition, *F*(2, 138) = 34.7, *p* < .001, ƞ_p_^2^ = 0.33, in which the positive condition presented a greater positive valence than either the negative (*p* < .001) or ambiguous (*p* < .001) conditions, and the ambiguous condition presented a greater positive valence than the negative (*p* < .001) condition. A Sex × Condition interaction, *F*(2, 138) = 3.3, *p* = .039, ƞ_p_^2^ = 0.05, was also identified, in which men showed a lower positive score in response to the positive condition than did women (*p* = .006). Finally, an Avoidance *×* Condition interaction, *F*(2, 138) = 10.8, *p* < .001, ƞ_p_^2^ = 0.13, was found.

The correlation analyses (Pearson’s *r*) performed between ECR-R scores (anxiety and avoidance) and the reaction times and emotional valence assignment of the visual stimuli revealed the following results. The ECR-R anxiety score did not show any significant correlations with either the reaction times or the emotional valence assignment of the visual stimuli. The ECR-R avoidance score was negatively correlated with the positive valence assignment of the visual stimuli (*r* = −0.49, *p* < .001). Coherently, the multiple factorial regression on the emotional valence assignment in response to the positive condition, with age, sex, ECR-R-anxiety score, ECR-R-avoidance score, and the Sex *×* ECR-R Score interaction as predictors showed a significant model. The sex and avoidance resulted as significant predictors (see Table 2).

**Table 2:** Multiple factorial regression analyses with age, sex, ECR-R-anxiety score, ECR-R-avoidance score, and the Sex × ECR-R Score interaction as predictors performed on the reaction times and the emotional valence assignment of the visual stimuli (positive, negative, and ambiguous conditions) (*n* = 74)

	Condition	Regression model	Predictors					
			Age	Sex	Anxiety	Sex × Anxiety	Avoidance	Sex × Avoidance
Reaction times	Positive	*R* = .16; *R*^2^ = .03; *R*^2^_adj_ = −.06 *F*(6, 67) = 0.30; *p* = .933; *SE* = 586.15	β = .04; *SE* = .12; *t*(67) = .35; *p* = .725	β = .10; *SE* = .12; *t*(67) = .78; *p* = .439	β = .11; *SE* = .14; *t*(67) = .80; *p* = .427	β = .11; *SE* = .13; *t*(67) = .80; *p* = .424	β = −.07; *SE* = .13; *t*(67) = −.52; *p* = .606	β = −.04; *SE* = .13; *t*(67) = −.33; *p* = .739
	Negative	*R* = .19; *R*^2^ = .03; *R*^2^_adj_ = −.05 *F*(6, 67) = 0.41; *p* = .867; *SE* = 659.12	β = −.05; *SE* = .12; *t*(67) = −.43; *p* = .670	β = .04; *SE* = .12; *t*(67) = .37; *p* = .715	β = −.04; *SE* = .13; *t*(67) = −.33; *p* = .742	β = .14; *SE* = .13; *t*(67) = 1.05; *p* = .294	β = −.07; *SE* = .12; *t*(67) = −.57; *p* = .570	β = −.03; *SE* = .12; *t*(67) = −.28; *p* = .781
	Ambiguous	*R* = .18; *R*^2^ = .03; *R*^2^_adj_ = −.05 *F*(6, 67) = 0.37; *p* = .893; *SE* = 778.89	β = .02; *SE* = .12; *t*(67) = .15; *p* = .878	β = −.04; *SE* = .12; *t*(67) = −.31; *p* = .758	β = .05; *SE* = .13; *t*(67) = .35; *p* = .729	β = .15; *SE* = .13; *t*(67) = 1.11; *p* = .267	β = −.11; *SE* = .13; *t*(67) = −.84; *p* = .405	β = −.07; *SE* = .13; *t*(67) = −.56; *p* = .574
Emotional valence assignment	Positive	***R*** **= .55;** ***R***^**2**^ **= .30;** ***R***^**2**^_**adj**_ **= .24** ***F*(6, 67) = 4.82;** ***p*** **< .001;** ***SE*** **= 0.44**	β = −.03; *SE* = .10; *t*(67) = −.27; *p* = .787	**β = −.24;** ***SE*** **= .10;** ***t*(67) = −2.34;** ***p*** **= .022**	β = .03; *SE* = .11; *t*(67) = .26; *p* = .799	β = .02; *SE* = .11; *t*(67) = .14; *p* = .887	**β = −.45;** ***SE*** **= .11;** ***t*(67) = −4.19;** ***p*** **< .001**	β = .01; *SE* = .11; *t*(67) = .13; *p* = .899
	Negative	*R* = .24; *R*^2^ = .06; *R*^2^_adj_ = −.03 *F*(6, 67) = 0.67; *p* = .676; *SE* = 0.51	β = .03; *SE* = .12; *t*(67) = .21; *p* = .833	β = .05; *SE* = .12; *t*(67) = .38; *p* = .707	β = −.13; *SE* = .13; *t*(67) = −.98; *p* = .331	β = .01; *SE* = .13; *t*(67) = .10; *p* = .920	β = .22; *SE* = .13 *t*(67) = 1.72; *p* = .090	β = −.01; *SE* = .12; *t*(67) = −.11; *p* = .913
	Ambiguous	*R* = .19; *R*^2^ = .04; *R*^2^_adj_ = −.05 *F*(6, 67) = 0.44; *p* = .850; *SE* = 0.56	β = −.06; *SE* = .12; *t*(67) = −.52; *p* = .601	β = .08; *SE* = .12; *t*(67) = .61; *p* = .540	β = .07; *SE* = .13; *t*(67) = .50; *p* = .620	β = .18; *SE* = .13; *t*(67) = 1.36; *p* = .179	β = −.06; *SE* = .12; *t*(67) = −.50; *p* = .618	β = −.05; *SE* = .12; *t*(67) = −.37; *p* = .711

*p* value < .05

### ERPs

**Fig. 2:**
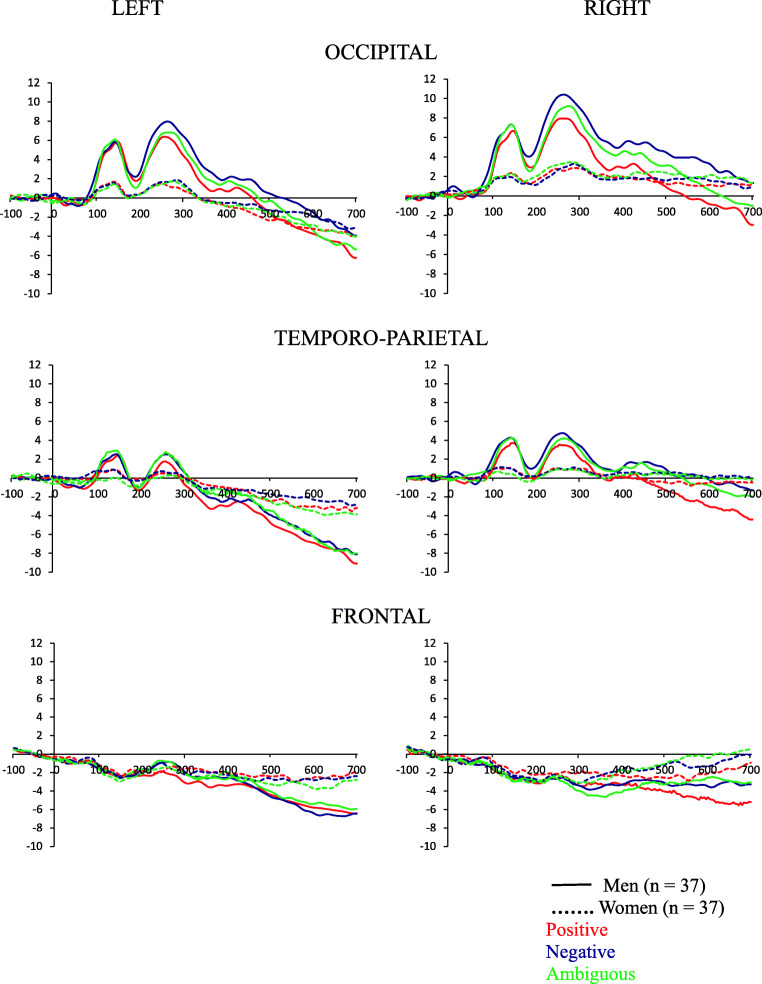
ERPs grand average of the left and right occipital, temporo-parietal, and frontal montages in response to positive, negative, and ambiguous conditions in men and women. (*n* = 74)

To analyze ERP data, the 2 (sex: men vs*.* women) × 3 (condition: positive vs. negative vs. ambiguous) × 2 (hemisphere: left vs. right) repeated-measures ANCOVAs, with age and ECR-R scores (anxiety and avoidance) as covariates, were performed on ERP amplitude (see Table 3 and Fig. 2). A main effect of sex was found on the occipital montage at P100 and on the temporo-parietal montage at P100 and P250, in which men showed a greater amplitude compared with those in women, and on the frontal montage at LC2, where men showed a lower amplitude than women. A Sex *×* Condition interaction was identified for the temporo-parietal montage at P100, in which men showed a greater amplitude in response to negative and ambiguous stimuli compared with women. In addition, ambiguous stimuli elicited a greater amplitude in men compared with positive stimuli, whereas in women this effect was inverted. A Sex *×* Condition *×* Hemisphere interaction was observed for the temporo-parietal montage at LC2. An Anxiety *×* Condition interaction was observed for the occipital montage at P100 and for the temporo-parietal montage at N200, P250, and LC1. An Avoidance × Condition interaction was identified for the occipital montage at P100. An Avoidance × Hemisphere interaction was observed for the temporo-parietal montage at P250. An Avoidance × Condition × Hemisphere interaction was found on the temporo-parietal montage at P250 and LC1 and on the frontal montage at P250. All the effects, also the not significant ones, were reported in Table 1 of the supplementary material (see Table 1s).

**Table 3:** The ANCOVAs sex [men (M) vs*.* women (W)] per condition [positive (Posi) vs*.* negative (Nega) vs. ambiguous (Ambi)] per hemisphere [left (_L_) vs. right (_R_)] performed on the amplitude and the latency of each event-related potential (ERP) component (P100, N200, P250, LC1, and LC2) on the occipital, temporo-parietal, and frontal montages, with age and the ECR-R scores (anxiety and avoidance) as covariates (*n* = 74)

ERP components	Significant effects in **montage** on amplitude and *latency*	Post hoc
P100 (80–160)	**Occipital**	
	Sex *F*(1, 69) = 16.5; *p* < .001; ƞ_p_^2^ = 0.19 Anxiety × Condition, *F*(2, 138) = 3.2; *p* = .042; ƞ_p_^2^ = 0.04 Avoidance × Condition, *F*(2, 138) = 3.4; *p* = .037; ƞ_p_^2^ = 0.05 Condition × Age, *F*(2, 138) = 4.1; *p* = .019; ƞ_p_^2^ = 0.06	Men > Women
	*Sex, F(1, 69) = 7.7; p = .007; ƞ*_*p*_^*2*^ *= 0.10*	*Men > Women*
	**Temporo-parietal**	
	Sex, *F*(1, 69) = 9.8; *p* = .002; ƞ_p_^2^ = 0.12	Men > Women
	Sex × Condition, *F*(2, 138) = 3.4; *p* = .036; ƞ_p_^2^ = 0.05 Condition × Hemisphere × Age, *F*(2, 138) = 3.7; *p* = .027; ƞ_p_^2^ = 0.05	MNega > WNega *p* = .007; MAmbi > WAmbi *p* < .001; MPosi < MNega *p* = .026; M*P*osi < MAmbi *p* = .037; W*P*osi > WAmbi *p* = .032; WNega > WAmbi *p* = .011
	*Sex, F(1, 69) = 22.9; p < .001; ƞ*_*p*_^*2*^ *= 0.25* *Anxiety × Condition × Hemisphere, F(2, 138) = 4.2; p = .018; ƞ*_*p*_^*2*^ *= 0.06*	*Men > Women*
N200 (160–220)	**Temporo-parietal**	
	Anxiety × Condition, *F*(2, 138) = 3.3; *p* = .039; ƞ_p_^2^ = 0.05 Condition × Hemisphere, *F*(2, 138) = 4.0; *p* = .021; ƞ_p_^2^ = 0.05 Condition × Age, *F*(2, 138) = 3.6; *p* = .031; ƞ_p_^2^ = 0.05 Condition × Hemisphere × Age, *F*(2, 138) = 3.9; *p* = .023; ƞ_p_^2^ = 0.05	Posi_R_ < Nega_R_ *p* = .011; Nega_R_ > Ambi_R_ *p* = .001; Posi_L_ < Posi_R_ *p* < .001; Nega_L_ < Nega_R_ *p* < .001; Ambi_L_ < Ambi_R_ *p* < .001
P250 (220–300)	**Temporo-parietal**	
	Sex, *F*(1, 69) = 4.2; *p* = .045; ƞ_p_^2^ = 0.06 Anxiety × Condition, *F*(2, 138) = 4.1; *p* = .018; ƞ_p_^2^ = 0.06 Avoidance × Hemisphere, *F*(1, 69) = 4.0; *p* = .049; ƞ_p_^2^ = 0.06 Condition × Hemisphere, *F*(2, 138) = 6.8; *p*= .002; ƞ_p_^2^ = 0.09 Avoidance × Condition × Hemisphere, *F*(2, 138) = 5.0; *p* = .008; ƞ_p_^2^ = 0.07 Condition × Hemisphere × Age, *F*(2, 138) = 8.3; *p* < .001; ƞ_p_^2^ = 0.11	Men > Women Posi_L_ < Nega_L_ *p* = .002; Posi_L_ < Ambi_L_ *p* = .025; Posi_R_ < Nega_R_ *p* = .002; Posi_L_ < Posi_R_ *p* < .001; Nega_L_ < Nega_R_ *p* < .001; Ambi_L_ < Ambi_R_ *p* < .001
	**Frontal**	
	Avoidance × Condition × Hemisphere, *F*(2, 138) = 3.3; *p* = .041; ƞ_p_^2^= 0.05 *Age, F(1, 69) = 4.4; p = .040; ƞ*_*p*_^*2*^ *= 0.06*	
LC1 (300–500)	**Temporo-parietal**	
	Anxiety × Condition, *F*(2, 138) = 3.6; *p* = .030; ƞ_p_^2^ = 0.05 Avoidance × Condition × Hemis*p*here, *F*(2, 138) = 3.1; *p* = .047; ƞ_p_^2^ = 0.04 Condition × Hemis*p*here × Age, *F*(2, 138) = 5.2; *p* = .007; ƞ_p_^2^ = 0.07	
LC2 (500–700)	**Temporo-parietal**	
	Sex × Condition × Hemisphere, *F*(2, 138) = 3.1; *p* = .048; ƞ_p_^2^ = 0.04	MPosi_R_ < MNega_R_ *p* < .001; MPosi_R_ < MAmbi_R_ *p* < .001; WNega_L_ > WAmbi_L_ *p* = .010; MPosi_L_ < MPosi_R_ *p* < .001; MNega_L_ < MNega_R_ *p* < .001; MAmbi_L_ < MAmbi_R_ *p* < .001; WPosi_L_ < WPosi_R_ *p* < .001; WNega_L_ < WNega_R_ *p* < .001; WAmbi_L_ < WAmbi_R_ *p* < .001
	**Frontal**	
	Sex, *F*(1, 69) = 4.0; *p* = .048; ƞ_p_^2^ = 0.06	Men < Women

ANCOVAs (sex: men vs. women) × (condition: positive vs. negative vs. ambiguous) × hemisphere (left vs. right), with age and the ECR-R scores (anxiety and avoidance) as covariates performed on the latency, showed the following results (see Table 3 and Fig. 2). A main effect of sex was observed for the occipital and temporo-parietal montages at P100, in which men showed a longer latency compared than women. A main effect of age was identified for the frontal montage at P250, in which age was negatively associated with the latency. An Anxiety × Condition × Hemisphere interaction was found on the temporo-parietal montage at P100.

### sLORETA

The correlation analyses performed between the ECR-R scores (anxiety and avoidance) and the mean intensity of each BA in each ROI in response to each condition, for each ERP component, is discussed below.

The ECR-R anxiety score did not show significant correlations with the brain intensity of any ROI, for any component (see Table 4). The ECR-R avoidance score was significantly positively correlated with the brain intensity of all ROIs in all components (see Table 4): for the early components (P100 and N200), the ECR-R avoidance scores correlated with brain intensity observed in the limbic, cingulate, and prefrontal cortices, mainly in response to negative and ambiguous conditions (see Table 4); for the late components (P250, LC1, and LC2), ECR-R avoidance scores correlated with the intensity of the limbic ROI in response to all conditions, with the intensity of the cingulate cortices in response to negative and ambiguous conditions, and with the intensity of the prefrontal cortex exclusively in response to negative condition (see Table 4).

**Table 4:** Correlations (Pearson’s *r*) performed between the ECR-R scores (anxiety and avoidance) and the mean intensity of the left (l) and right (r) Brodmann areas (BAs) for each region of interest (ROI), including the limbic ROI [amygdala (AMG), insula, amygdala-hippocampus junction (AHj), and hippocampus (HPC)], the anterior cingulate cortex ROI (BA24, BA32, and BA33), the posterior cingulate cortex ROI (BA23, BA30, and BA31), and the prefrontal cortex ROI (BA09, BA10, BA11, BA46, and BA47), for the three conditions (positive, negative, and ambiguous) on the event-related potential (ERP) components (P100, N200, P250, LC1, and LC2) (Bonferroni correction was applied with accepted *p* value ≤ .0003) (*n* = 74)

ERP	ROI	Positive condition		Negative condition		Ambiguous condition	
	BA	Anxiety	Avoidance	Anxiety	Avoidance	Anxiety	Avoidance
P100	Limbic						
(80–160)	lAMG	*r* = .16; *p* = .1809	*r* = .20; *p* = .0836	*r* = .10; *p* = .4005	*r* = .32; *p* = .0056	*r* = .09; *p* = .4537	*r* = .29; *p* = .0111
	rAMG	*r* = .21; *p* = .0689	***r*** **= .41;** ***p*** **= .0003**	*r* = .22; *p* = .0548	***r*** **= .41;** ***p*** **= .0003**	*r* = .23; *p* = .0513	***r*** **= .45;** ***p*** **= .0001**
	lInsula	*r* = .26; *p* = .0266	*r* = .27; *p* = .0193	*r* = .14; *p* = .2193	*r* = .27; *p* = .0195	*r* = .19; *p* = .1072	*r* = .30; *p* = .0094
	rInsula	*r* = .18; *p* = .1253	*r* = .36; *p* = .0014	*r* = .22; *p* = .0634	*r* = .36; *p* = .0019	*r* = .22; *p* = .0600	*r* = .40; *p* = .0004
	lAHj	*r* = 18; *p* = .1174	*r* = .27; *p* = .0195	*r* = .13; *p* = .2704	*r* = .37; *p* = .0011	*r* = .13; *p* = .2869	*r* = .35; *p* = .0021
	rAHj	*r* = 21; *p* = .0663	***r*** **= .41;** ***p*** **= .0003**	*r* = .22; *p* = .0647	***r*** **= .42;** ***p*** **= .0002**	*r* = .23; *p* = .0501	***r*** **= .44;** ***p*** **= .0001**
	lHPC	*r* = .17; *p* = .1490	*r* = .18; *p* = .1198	*r* = .11; *p* = .3647	*r* = .26; *p* = .0255	*r* = .13; *p* = .2574	*r* = .27; *p* = .0201
	rHPC	*r* = .27; *p* = .0202	***r*** **= .41;** ***p*** **= .0002**	*r* = .27; *p* = .0197	*r* = .38; *p* = .0008	*r* = .30; *p* = .0083	***r*** **= .43;** ***p*** **= .0001**
	ACC						
	lBA24	*r* = .22; *p* = .0646	*r* = .26; *p* = .0252	*r* = .18; *p* = .1328	*r* = .31; *p* = .0077	*r* = .21; *p* = .0751	*r* = .30; *p* = .0104
	rBA24	*r* = .19; *p* = .1055	*r* = .29; *p* = .0123	*r* = .16; *p* = .1730	*r* = .33; *p* = .0039	*r* = .19; *p* = .1077	*r* = .30; *p* = .0103
	lBA32	*r* = .21; *p* = .0738	*r* = .36; *p* = .0019	*r* = .20; *p* = .0819	***r*** **= .43;** ***p*** **= .0001**	*r* = .23; *p* = .0537	***r*** **= .42;** ***p*** **= .0002**
	rBA32	*r* = .19; *p* = .0984	*r* = .35; *p* = .0020	*r* = .20; *p* = .0859	***r*** **= .41;** ***p*** **= .0003**	*r* = .24; *p* = .0426	***r*** **= .44;** ***p*** **= .0001**
	lBA33	*r* = .22; *p* = .0608	*r* = .33; *p* = .0036	*r* = .20; *p* = .0933	*r* = .39; *p* = .0006	*r* = .22; *p* = .0558	*r* = .39; *p* = .0006
	rBA33	*r* = .21; *p* = .0684	*r* = .34; *p* = .0031	*r* = .20; *p* = .0906	*r* = .38; *p* = .0008	*r* = .23; *p* = .0503	*r* = .39; *p* = .0005
	PCC						
	lBA23	*r* = .19; *p* = .1065	*r* = .34; *p* = .0034	*r* = .13; *p* = .2563	*r* = .38; *p* = .0010	*r* = .13; *p* = .2634	*r* = .39; *p* = .0005
	rBA23	*r* = .19; *p* = .1045	*r* = .40; *p* = .0005	*r* = .15; *p* = .2038	***r*** **= .41;** ***p*** **= .0003**	*r* = .12; *p* = .3207	***r*** **= .41;** ***p*** **= .0003**
	lBA30	*r* = .23; *p* = .0504	*r* = .29; *p* = .0138	*r* = .15; *p* = .2153	*r* = .33; *p* = .0043	*r* = .19; *p* = .0974	*r* = .36; *p* = .0016
	rBA30	*r* = .27; *p* = .0196	*r* = .39; *p* = .0006	*r* = .22; *p* = .0641	*r* = .40; *p* = .0004	*r* = .24; *p* = .0390	***r*** **= .41;** ***p*** **= .0003**
	lBA31	*r* = .14; *p* = .2212	*r* = .29; *p* = .0110	*r* = .09; *p* = .4264	*r* = .35; *p* = .0024	*r* = .04; *p* = .7634	*r* = .35; *p* = .0021
	rBA31	*r* = .12; *p* = .3257	*r* = .37; *p* = .0014	*r* = .08; *p* = .5054	*r* = .40; *p* = .0005	*r* = −.01; *p* = .9617	*r* = .36; *p* = .0015
	PFC						
	lBA09	*r* = .28; *p* = .0144	*r* = .33; *p* = .0040	*r* = .25; *p* = .0342	*r* = .39; *p* = .0005	*r* = .28; *p* = .0141	*r* = .37; *p* = .0014
	rBA09	*r* = .17; *p* = .1818	*r* = .29; *p* = .0125	*r* = .16; *p* = .1792	*r* = .30; *p* = .0093	*r* = .13; *p* = .2406	*r* = .32; *p* = .0061
	lBA10	*r* = .18; *p* = .1289	*r* = .26; *p* = .0259	*r* = .14; *p* = .2339	*r* = .30; *p* = .0096	*r* = .22; *p* = .0631	*r* = .31; *p* = .0071
	rBA10	*r* = .19; *p* = .1010	*r* = .19; *p* = .1074	*r* = .17; *p* = .1422	*r* = .25; *p* = .0316	*r* = .15; *p* = .1910	*r* = .26; *p* = .0265
	lBA11	*r* = .18; *p* = .1160	*r* = .35; *p* = .0024	*r* = .16; *p* = .1777	***r*** **= .43;** ***p*** **= .0001**	*r* = .23; *p* = .0492	***r*** **= .41;** ***p*** **= .0003**
	rBA11	*r* = .13; *p* = .2599	*r* = .34; *p* = .0035	*r* = .15; *p* = .2075	*r* = .37; *p* = .0007	*r* = .16; *p* = .1850	***r*** **= .42;** ***p*** **= .0002**
	lBA46	*r* = .28; *p* = .0153	*r* = .34; *p* = .0027	*r* = .23; *p* = .0495	*r* = .35; *p* = .0021	*r* = .25; *p* = .0300	*r* = .40; *p* = .0004
	rBA46	*r* = .06; *p* = .6099	*r* = .19; *p* = .1068	*r* = .07; *p* = .5358	*r* = .21; *p* = .0789	*r* = .05; *p* = .6726	*r* = .19; *p* = .1124
	lBA47	*r* = .23; *p* = .0480	*r* = .32; *p* = .0061	*r* = .16; *p* = .1836	*r* = .33; *p* = .0036	*r* = .20; *p* = .0820	*r* = .32; *p* = .0049
	rBA47	*r* = −.01; *p* = .9074	*r* = .35; *p* = .0025	*r* = .02; *p* = .8517	*r* = .35; *p* = .0020	*r* = .00; *p* = .9894	*r* = .40; *p* = .0004
N200	Limbic						
(160–220)	lAMG	*r* = .10; *p* = .3804	*r* = .26; *p* = .0255	*r* = .09; *p* = .4533	*r* = .28; *p* = .0147	*r* = .06; *p* = .6206	*r* = .30; *p* = .0104
	rAMG	*r* = .22; *p* = .0646	***r*** **= .43;** ***p*** **= .0001**	*r* = .18; *p* = .1366	***r*** **= .43;** ***p*** **= .0001**	*r* = .22; *p* = .0542	***r*** **= .43;** ***p*** **= .0001**
	lInsula	*r* = .20; *p* = .0885	*r* = .31; *p* = .0070	*r* = .12; *p* = .3123	*r* = .28; *p* = .0159	*r* = .16; *p* = .1764	*r* = .29; *p* = .0121
	rInsula	*r* = .19; *p* = .1088	*r* = .37; *p* = .0012	*r* = .19; *p* = .1041	*r* = .36; *p* = .0017	*r* = .23; *p* = .0512	*r* = .39; *p* = .0007
	lAHj	*r* = .13; *p* = .2628	*r* = .34; *p* = .0033	*r* = .12; *p* = .3056	*r* = .35; *p* = .0023	*r* = .10; *p* = .4012	*r* = .34; *p* = .0027
	rAHj	*r* = .22; *p* = .0641	***r*** **= .44;** ***p*** **= .0001**	*r* = .18; *p* = .1299	***r*** **= .44;** ***p*** **= .0001**	*r* = .22; *p* = .0647	***r*** **= .42;** ***p*** **= .0002**
	lHPC	*r* = .13; *p* = .2740	*r* = .21; *p* = .0673	*r* = .07; *p* = .5606	*r* = .24; *p* = .0435	*r* = .09; *p* = .4415	*r* = .25; *p* = .0327
	rHPC	*r* = .26; *p* = .0250	*r* = .40; *p* = .0004	*r* = .22; *p* = .0568	***r*** **= .41;** ***p*** **= .0003**	*r* = .30; *p* = .0101	***r*** **= .42;** ***p*** **= .0002**
	ACC						
	lBA24	*r* = .21; *p* = .0797	*r* = .30; *p* = .0106	*r* = .13; *p* = .2875	*r* = .34; *p* = .0034	*r* = .16; *p* = .1789	*r* = .27; *p* = .0185
	rBA24	*r* = .18; *p* = .1224	*r* = .32; *p* = .0048	*r* = .12; *p* = .3198	*r* = .38; *p* = .0009	*r* = .15; *p* = .1910	*r* = .27; *p* = .0205
	lBA32	*r* = .20; *p* = .0849	*r* = .37; *p* = .0011	*r* = .15; *p* = .1953	***r*** **= .43;** ***p*** **= .0001**	*r* = .21; *p* = .0682	*r* = .39; *p* = .0005
	rBA32	*r* = .21; *p* = .0772	*r* = .39; *p* = .0005	*r* = .16; *p* = .1671	***r*** **= .44;** ***p*** **= .0001**	*r* = .20; *p* = .0926	*r* = .38; *p* = .0008
	lBA33	*r* = .22; *p* = .0641	*r* = .37; *p* = .0013	*r* = .15; *p* = .1970	*r* = .39; *p* = .0005	*r* = .20; *p* = .0875	*r* = .36; *p* = .0016
	rBA33	*r* = .23; *p* = .0509	*r* = .38; *p* = .0009	*r* = .16; *p* = .1715	***r*** **= .41;** ***p*** **= .0003**	*r* = .21; *p* = .0729	*r* = .35; *p* = .0021
	PCC						
	lBA23	*r* = .15; *p* = .2048	*r* = .36; *p* = .0016	*r* = .09; *p* = .4247	*r* = .37; *p* = .0011	*r* = .09; *p* = .4528	*r* = .37; *p* = .0014
	rBA23	*r* = .18; *p* = .1352	***r*** **= .41;** ***p*** **= .0003**	*r* = .13; *p* = .2724	*r* = .38; *p* = .0007	*r* = .08; *p* = .4790	*r* = .40; *p* = .0004
	lBA30	*r* = .19; *p* = .1045	*r* = .32; *p* = .0060	*r* = .10; *p* = .4149	*r* = .33; *p* = .0044	*r* = .16; *p* = .1690	*r* = .33; *p* = .0044
	rBA30	*r* = .24; *p* = .0360	*r* = .39; *p* = .0007	*r* = .19; *p* = .1001	*r* = .36; *p* = .0016	*r* = .23; *p* = .0483	*r* = .40; *p* = .0004
	lBA31	*r* = .10; *p* = .4145	*r* = .33; *p* = .0044	*r* = .05; *p* = .6515	*r* = .35; *p* = .0021	*r* = −.01; *p* = .9028	*r* = .33; *p* = .0040
	rBA31	*r* = .11; *p* = .3566	*r* = .40; *p* = .0005	*r* = .07; *p* = .5802	*r* = .38; *p* = .0009	*r* = −.04; *p* = .7593	*r* = .36; *p* = .0017
	PFC						
	lBA09	*r* = .23; *p* = .0445	*r* = .35; *p* = .0023	*r* = .24; *p* = .0408	*r* = .39; *p* = .0006	*r* = .27; *p* = .0196	*r* = .34; *p* = .0028
	rBA09	*r* = .16; *p* = .1843	*r* = .33; *p* = .0039	*r* = .11; *p* = .3328	*r* = .34; *p* = .0030	*r* = .12; *p* = .2965	*r* = .30; *p* = .0086
	lBA10	*r* = .17; *p* = .1438	*r* = .29; *p* = .0108	*r* = .12; *p* = .3258	*r* = .30; *p* = .0104	*r* = .18; *p* = .1257	*r* = .29; *p* = .0122
	rBA10	*r* = .19; *p* = .1026	*r* = .23; *p* = .0537	*r* = .15; *p* = .2044	*r* = .26; *p* = .0227	*r* = .15; *p* = .1949	*r* = .20; *p* = .0827
	lBA11	*r* = .19; *p* = .1149	***r*** **= .42;** ***p*** **= .0002**	*r* = .13; *p* = .2728	***r*** **= .43;** ***p*** **= .0001**	*r* = .21; *p* = .0724	*r* = .38; *p* = .0007
	rBA11	*r* = .12; *p* = .2945	*r* = .40; *p* = .0004	*r* = .13; *p* = .2628	***r*** **= .41;** ***p*** **= .0003**	*r* = .13; *p* = .2837	*r* = .38; *p* = .0007
	lBA46	*r* = .24; *p* = .0408	*r* = .38; *p* = .0008	*r* = .21; *p* = .0688	*r* = .34; *p* = .0029	*r* = .25; *p* = .0306	*r* = .36; *p* = .0018
	rBA46	*r* = .08; *p* = .5221	*r* = .24; *p* = .0378	*r* = .04; *p* = .7632	*r* = .22; *p* = .0586	*r* = .06; *p* = .5868	*r* = .22; *p* = .0602
	lBA47	*r* = .20; *p* = .0905	*r* = .38; *p* = .0008	*r* = .14; *p* = .2400	*r* = .34; *p* = .0031	*r* = .18; *p* = .1233	*r* = .30; *p* = .0102
	rBA47	*r* = .01; *p* = .9185	*r* = .38; *p* = .0007	*r* = .03; *p* = .8025	*r* = .37; *p* = .0014	*r* = .01; *p* = .9362	*r* = .37; *p* = .0012
P250	Limbic						
(220–300)	lAMG	*r* = .15; *p* = .1928	*r* = .26; *p* = .0233	*r* = .06; *p* = .6140	*r* = .29; *p* = .0135	*r* = .11; *p* = .3673	*r* = .28; *p* = .0152
	rAMG	*r* = .21; *p* = .0733	***r*** **= .43;** ***p*** **= .0001**	*r* = .20; *p* = .0851	***r*** **= .42;** ***p*** **= .0002**	*r* = .22; *p* = .0604	***r*** **= .43;** ***p*** **= .0001**
	lInsula	*r* = .25; *p* = .0353	*r* = .31; *p* = .0072	*r* = .13; *p* = .2821	*r* = .28; *p* = .0146	*r* = .18; *p* = .1161	*r* = .28; *p* = .0151
	rInsula	*r* = .18; *p* = .1283	*r* = .39; *p* = .0007	*r* = .16; *p* = .1746	*r* = .36; *p* = .0015	*r* = .22; *p* = .0591	*r* = .38; *p* = .0010
	lAHj	*r* = .16; *p* = .1692	*r* = .32; *p* = .0055	*r* = .10; *p* = .4173	*r* = .34; *p* = .0031	*r* = .13; *p* = .2645	*r* = .34; *p* = .0034
	rAHj	*r* = .20; *p* = .0809	***r*** **= .43;** ***p*** **= .0001**	*r* = .19; *p* = .0982	***r*** **= .43;** ***p*** **= .0002**	*r* = .22; *p* = .0654	***r*** **= .43;** ***p*** **= .0001**
	lHPC	*r* = .18; *p* = .1190	*r* = .23; *p* = .0490	*r* = .08; *p* = .4961	*r* = .25; *p* = .0311	*r* = .16; *p* = .1779	*r* = .26; *p* = .0265
	rHPC	*r* = .26; *p* = .0274	***r*** **= .42;** ***p*** **= .0002**	*r* = .25; *p* = .0295	*r* = .40; *p* = .0004	*r* = .31; *p* = .0064	***r*** **= .41;** ***p*** **= .0003**
	ACC						
	lBA24	*r* = .20; *p* = .0955	*r* = .29; *p* = .0112	*r* = .18; *p* = .1315	*r* = .36; *p* = .0015	*r* = .18; *p* = .1185	*r* = .26; *p* = .0279
	rBA24	*r* = .18; *p* = .1177	*r* = .29; *p* = .0120	*r* = .14; *p* = .2362	***r*** **= .41;** ***p*** **= .0003**	*r* = .18; *p* = .1303	*r* = .26; *p* = .0228
	lBA32	*r* = .22; *p* = .0650	*r* = .40; *p* = .0004	*r* = .19; *p* = .1151	***r*** **= .45;** ***p*** **= .0001**	*r* = .22; *p* = .0579	***r*** **= .41;** ***p*** **= .0003**
	rBA32	*r* = .23; *p* = .0519	*r* = .38; *p* = .0009	*r* = .19; *p* = .1134	***r*** **= .45;** ***p*** **= .0001**	*r* = .22; *p* = .0618	*r* = .40; *p* = .0004
	lBA33	*r* = .21; *p* = .0679	*r* = .37; *p* = .0012	*r* = .18; *p* = .1319	*r* = .40; *p* = .0004	*r* = .22; *p* = .0606	*r* = .36; *p* = .0017
	rBA33	*r* = .22; *p* = .0635	*r* = .37; *p* = .0014	*r* = .18; *p* = .1243	*r* = .40; *p* = .0004	*r* = .22; *p* = .0558	*r* = .35; *p* = .0023
	PCC						
	lBA23	*r* = .14; *p* = .2207	*r* = .36; *p* = .0017	*r* = .12; *p* = .3263	*r* = .38; *p* = .0008	*r* = .12; *p* = .3277	*r* = .38; *p* = .0009
	rBA23	*r* = .16; *p* = .1797	*r* = .40; *p* = .0004	*r* = .14; *p* = .2486	***r*** **= .42;** ***p*** **= .0002**	*r* = .12; *p* = .3259	***r*** **= .41;** ***p*** **= .0003**
	lBA30	*r* = .22; *p* = .0649	*r* = .32; *p* = .0061	*r* = .13; *p* = .2763	*r* = .33; *p* = .0036	*r* = .20; *p* = .0899	*r* = .35; *p* = .0021
	rBA30	*r* = .26; *p* = .0262	*r* = .39; *p* = .0005	*r* = .21; *p* = .0753	*r* = .39; *p* = .0006	*r* = .26; *p* = .0228	*r* = .39; *p* = .0005
	lBA31	*r* = .08; *p* = .4729	*r* = .33; *p* = .0039	*r* = .07; *p* = .5730	*r* = .35; *p* = .0023	*r* = .02; *p* = .8403	*r* = .34; *p* = .0036
	rBA31	*r* = .08; *p* = .5061	*r* = .39; *p* = .0007	*r* = .07; *p* = .5331	*r* = .40; *p* = .0005	*r* = −.02; *p* = .8589	*r* = .36; *p* = .0015
	PFC						
	lBA09	*r* = .26; *p* = .0279	*r* = .38; *p* = .0008	*r* = .21; *p* = .0673	*r* = .37; *p* = .0011	*r* = .27; *p* = .02003	*r* = .35; *p* = .0022
	rBA09	*r* = .16; *p* = .1872	*r* = .30; *p* = .0094	*r* = .10; *p* = .4019	*r* = .32; *p* = .0051	*r* = .14; *p* = .2489	*r* = .28; *p* = .0153
	lBA10	*r* = .21; *p* = .0757	*r* = .32; *p* = .0048	*r* = .14; *p* = .2482	*r* = .29; *p* = .0125	*r* = .18; *p* = .1285	*r* = .29; *p* = .0131
	rBA10	*r* = .21; *p* = .0735	*r* = .24; *p* = .0393	*r* = .16; *p* = .1737	*r* = .26; *p* = .0282	*r* = .17; *p* = .1601	*r* = .21; *p* = .0784
	lBA11	*r* = .19; *p* = .1017	***r*** **= .43;** ***p*** **= .0001**	*r* = .16; *p* = .1806	***r*** **= .44;** ***p*** **= .0001**	*r* = .20; *p* = .0848	*r* = .40; *p* = .0004
	rBA11	*r* = .14; *p* = .2250	*r* = .40; *p* = .0004	*r* = .10; *p* = .3872	*r* = .40; *p* = .0004	*r* = .12; *p* = .2997	*r* = .39; *p* = .0005
	lBA46	*r* = .26; *p* = .0284	*r* = .39; *p* = .0007	*r* = .19; *p* = .1075	*r* = .36; *p* = .0018	*r* = .23; *p* = .0440	*r* = .37; *p* = .0013
	rBA46	*r* = .05; *p* = .6517	*r* = .22; *p* = .0600	*r* = .01; *p* = .9222	*r* = .19; *p* = .1012	*r* = .05; *p* = .6960	*r* = .18; *p* = .1355
	lBA47	*r* = .21; *p* = .0673	*r* = .37; *p* = .0014	*r* = .14; *p* = .2266	*r* = .34; *p* = .0029	*r* = .18; *p* = .1192	*r* = .32; *p* = .0063
	rBA47	*r* = −.01; *p* = .9241	*r* = .37; *p* = .0012	*r* = −.04; *p* = .7499	*r* = .36; *p* = .0018	*r* = −.02; *p* = .8817	*r* = .37; *p* = .0011
LC1	Limbic						
(300–500)	lAMG	*r* = .15; *p* = .2036	*r* = .26; *p* = .0236	*r* = .08; *p* = .5018	*r* = .30; *p* = .0089	*r* = .11; *p* = .3545	*r* = .24; *p* = .0388
	rAMG	*r* = .23; *p* = .0509	***r*** **= .43;** ***p*** **= .0002**	*r* = .19; *p* = .0990	***r*** **= .45;** ***p*** **= .0001**	*r* = .24; *p* = .0430	***r*** **= .41;** ***p*** **= .0002**
	lInsula	*r* = .23; *p* = .0484	*r* = .29; *p* = .0198	*r* = .16; *p* = .1814	*r* = .31; *p* = .0066	*r* = .21; *p* = .0773	*r* = .28; *p* = .0169
	rInsula	*r* = .21; *p* = .0731	*r* = .37; *p* = .0013	*r* = .19; *p* = .1115	*r* = .40; *p* = .0004	*r* = .21; *p* = .0709	*r* = .35; *p* = .0024
	lAHj	*r* = .18; *p* = .1177	*r* = .33; *p* = .0041	*r* = .12; *p* = .2994	*r* = .37; *p* = .0012	*r* = .15; *p* = .2148	*r* = .29; *p* = .0126
	rAHj	*r* = .24; *p* = .0432	***r*** **= .43;** ***p*** **= .0002**	*r* = .20; *p* = .0928	***r*** **= .46;** ***p*** **= .0001**	*r* = .23; *p* = .0458	*r* = .40; *p* = .0004
	lHPC	*r* = .17; *p* = .1485	*r* = .23; *p* = .0507	*r* = .11; *p* = .3743	*r* = .27; *p* = .0211	*r* = .13; *p* = .2800	*r* = .21; *p* = .0665
	rHPC	*r* = .28; *p* = .0160	***r*** **= .42;** ***p*** **= .0002**	*r* = .25; *p* = .0322	***r*** **= .42;** ***p*** **= .0002**	*r* = .31; *p* = .0074	*r* = .40; *p* = .0005
	ACC						
	lBA24	*r* = .21; *p* = .0661	*r* = .28; *p* = .0166	*r* = .18; *p* = .1233	*r* = .34; *p* = .0033	*r* = .19; *p* = .1032	*r* = .25; *p* = .0318
	rBA24	*r* = .18; *p* = .1193	*r* = .30; *p* = .0095	*r* = .16; *p* = .1580	*r* = .37; *p* = .0010	*r* = .18; *p* = .1221	*r* = .25; *p* = .0298
	lBA32	*r* = .23; *p* = .0496	*r* = .38; *p* = .0008	*r* = .19; *p* = .1054	***r*** **= .45;** ***p*** **= .0001**	*r* = .24; *p* = .0367	*r* = .39; *p* = .0007
	rBA32	*r* = .23; *p* = .0472	*r* = .38; *p* = .0010	*r* = .20; *p* = .0832	***r*** **= .43;** ***p*** **= .0001**	*r* = .24; *p* = .0384	*r* = .38; *p* = .0009
	lBA33	*r* = .24; *p* = .0378	*r* = .36; *p* = .0018	*r* = .20; *p* = .0840	***r*** **= .41;** ***p*** **= .0003**	*r* = .23; *p* = .0525	*r* = .34; *p* = .0026
	rBA33	*r* = .24; *p* = .0392	*r* = .36; *p* = .0019	*r* = .21; *p* = .0796	***r*** **= .41;** ***p*** **= .0003**	*r* = .23; *p* = .0535	*r* = .34; *p* = .0032
	PCC						
	lBA23	*r* = .18; *p* = .1359	*r* = .36; *p* = .0014	*r* = .11; *p* = .3559	***r*** **= .41;** ***p*** **= .0003**	*r* = .10; *p* = .3972	*r* = .36; *p* = .0016
	rBA23	*r* = .18; *p* = .1245	*r* = .40; *p* = .0004	*r* = .14; *p* = .2506	***r*** **= .43;** ***p*** **= .0001**	*r* = .11; *p* = .3634	*r* = .39; *p* = .0005
	lBA30	*r* = .21; *p* = .0706	*r* = .33; *p* = .0042	*r* = .13; *p* = .2533	*r* = .37; *p* = .0012	*r* = .17; *p* = .1456	*r* = .32; *p* = .0052
	rBA30	*r* = .25; *p* = .0291	*r* = .39; *p* = .0006	*r* = .22; *p* = .0622	***r*** **= .41;** ***p*** **= .0003**	*r* = .25; *p* = .0303	*r* = .38; *p* = .0009
	lBA31	*r* = .12; *p* = .3276	*r* = .33; *p* = .0037	*r* = .06; *p* = .5979	*r* = .37; *p* = .0012	*r* = .01; *p* = .9325	*r* = .32; *p* = .0051
	rBA31	*r* = .11; *p* = .3344	*r* = .39; *p* = .0006	*r* = .06; *p* = .6195	***r*** **= .41;** ***p*** **= .0003**	*r* = −.02; *p* = .8984	*r* = .35; *p* = .0021
	PFC						
	lBA09	*r* = .25; *p* = .0302	*r* = .37; *p* = .0012	*r* = .26; *p* = .0283	*r* = .39; *p* = .0005	*r* = .27; *p* = .0199	*r* = .35; *p* = .0026
	rBA09	*r* = .14; *p* = .2389	*r* = .28; *p* = .0157	*r* = .13; *p* = .2732	*r* = .31; *p* = .0080	*r* = .14; *p* = .2241	*r* = .28; *p* = .0168
	lBA10	*r* = .19; *p* = .1129	*r* = .30; *p* = .0098	*r* = .14; *p* = .2272	*r* = .31; *p* = .0063	*r* = .20; *p* = .0893	*r* = .28; *p* = .0165
	rBA10	*r* = .18; *p* = .1212	*r* = .21; *p* = .0683	*r* = .18; *p* = .1282	*r* = .26; *p* = .0282	*r* = .16; *p* = .1708	*r* = .23; *p* = .0525
	lBA11	*r* = .21; *p* = .0723	*r* = .38; *p* = .0008	*r* = .13; *p* = .2898	***r*** **= .45;** ***p*** **= .0001**	*r* = .25; *p* = .0328	*r* = .37; *p* = .0012
	rBA11	*r* = .15; *p* = .2137	*r* = .36; *p* = .0015	*r* = .11; *p* = .3572	***r*** **= .41;** ***p*** **= .0003**	*r* = .16; *p* = .1692	*r* = .36; *p* = .0025
	lBA46	*r* = .26; *p* = .0274	*r* = .40; *p* = .0004	*r* = .21; *p* = .0785	*r* = .38; *p* = .0009	*r* = .28; *p* = .0176	*r* = .37; *p* = .0011
	rBA46	*r* = .06; *p* = .5865	*r* = .18; *p* = .1317	*r* = .03; *p* = .7861	*r* = .18; *p* = .1266	*r* = .05; *p* = .6935	*r* = .19; *p* = .1046
	lBA47	*r* = .23; *p* = .0531	*r* = .35; *p* = .0025	*r* = .13; *p* = .2591	*r* = .37; *p* = .0010	*r* = .22; *p* = .0572	*r* = .30; *p* = .0083
	rBA47	*r* = .01; *p* = .9342	*r* = .35; *p* = .0021	*r* = −.01; *p* = .9114	*r* = .37; *p* = .0012	*r* = −.00; *p* = .9800	*r* = .35; *p* = .0021
LC2	Limbic						
(500–700)	lAMG	*r* = .18; *p* = .1303	*r* = .24; *p* = .0432	*r* = .13; *p* = .2771	*r* = .31; *p* = .0065	*r* = .11; *p* = .3516	*r* = .26; *p* = .0267
	rAMG	*r* = .22; *p* = .0541	***r*** **= .43;** ***p*** **= .0001**	*r* = .23; *p* = .0492	***r*** **= .44;** ***p*** **= .0001**	*r* = .21; *p* = .0670	***r*** **= .44;** ***p*** **= .0001**
	lInsula	*r* = .25; *p* = .0327	*r* = .30; *p* = .0096	*r* = .19; *p* = .1056	*r* = .32; *p* = .0049	*r* = .19; *p* = .1014	*r* = .28; *p* = .0155
	rInsula	*r* = .21; *p* = .0700	*r* = .36; *p* = .0018	*r* = .19; *p* = .1091	*r* = .39; *p* = .0005	*r* = .18; *p* = .1198	*r* = .39; *p* = .0006
	lAHj	*r* = .21; *p* = .0799	*r* = .31; *p* = .0071	*r* = .17; *p* = .1611	*r* = .38; *p* = .0007	*r* = .15; *p* = .2097	*r* = .31; *p* = .0076
	rAHj	*r* = .23; *p* = .0478	***r*** **= .43;** ***p*** **= .0001**	*r* = .23; *p* = .0470	***r*** **= .45;** ***p*** **= .0001**	*r* = .22; *p* = .0620	***r*** **= .42;** ***p*** **= .0002**
	lHPC	*r* = .20; *p* = .0922	*r* = .22; *p* = .0557	*r* = .13; *p* = .2827	*r* = .28; *p* = .0177	*r* = .14; *p* = .2520	*r* = .24; *p* = .0409
	rHPC	*r* = .29; *p* = .0126	***r*** **= .42;** ***p*** **= .0002**	*r* = .27; *p* = .0199	***r*** **= .42;** ***p*** **= .0002**	*r* = .29; *p* = .0132	***r*** **= .42;** ***p*** **= .0002**
	ACC						
	lBA24	*r* = .22; *p* = .0559	*r* = .29; *p* = .0137	*r* = .20; *p* = .0950	*r* = .33; *p* = .0045	*r* = .18; *p* = .1327	*r* = .25; *p* = .0342
	rBA24	*r* = .18; *p* = .1208	*r* = .31; *p* = .0082	*r* = .18; *p* = .1244	*r* = .35; *p* = .0020	*r* = .17; *p* = .1605	*r* = .25; *p* = .0337
	lBA32	*r* = .25; *p* = .0330	*r* = .39; *p* = .0007	*r* = .21; *p* = .0739	***r*** **= .44;** ***p*** **= .0001**	*r* = .22; *p* = .0566	*r* = .38; *p* = .0007
	rBA32	*r* = .23; *p* = .0500	*r* = .39; *p* = .0006	*r* = .21; *p* = .0668	***r*** **= .43;** ***p*** **= .0001**	*r* = .21; *p* = .0760	*r* = .39; *p* = .0006
	lBA33	*r* = .26; *p* = .0268	*r* = .37; *p* = .0012	*r* = .23; *p* = .0525	***r*** **= .41;** ***p*** **= .0003**	*r* = .21; *p* = .0705	*r* = .35; *p* = .0022
	rBA33	*r* = .25; *p* = .0315	*r* = .37; *p* = .0011	*r* = .23; *p* = .0481	***r*** **= .41;** ***p*** **= .0003**	*r* = .22; *p* = .0657	*r* = .35; *p* = .0023
	PCC						
	lBA23	*r* = .17; *p* = .1485	*r* = .35; *p* = .0022	*r* = .11; *p* = .3401	***r*** **= .42;** ***p*** **= .0002**	*r* = .12; *p* = .3067	*r* = .39; *p* = .0006
	rBA23	*r* = .18; *p* = .1369	*r* = .40; *p* = .0004	*r* = .15; *p* = .2186	***r*** **= .44;** ***p*** **= .0001**	*r* = .12; *p* = .3287	***r*** **= .42;** ***p*** **= .0002**
	lBA30	*r* = .23; *p* = .0476	*r* = .32; *p* = .0062	*r* = .14; *p* = .2232	*r* = .38; *p* = .0010	*r* = .19; *p* = .1014	*r* = .35; *p* = .0020
	rBA30	*r* = .27; *p* = .0184	*r* = .39; *p* = .0007	*r* = .23; *p* = .0457	***r*** **= .41;** ***p*** **= .0003**	*r* = .24; *p* = .0404	***r*** **= .41;** ***p*** **= .0003**
	lBA31	*r* = .11; *p* = .3518	*r* = .32; *p* = .0053	*r* = .06; *p* = .5976	*r* = .38; *p* = .0008	*r* = .02; *p* = .8670	*r* = .35; *p* = .0024
	rBA31	*r* = .09; *p* = .4497	*r* = .38; *p* = .0008	*r* = .08; *p* = .5128	***r*** **= .42;** ***p*** **= .0002**	*r* = −.01; *p* = .9673	*r* = .37; *p* = .0010
	PFC						
	lBA09	*r* = .26; *p* = .0249	*r* = .36; *p* = .0014	*r* = .25; *p* = .0289	*r* = .39; *p* = .0006	*r* = .26; *p* = .0272	*r* = .34; *p* = .0029
	rBA09	*r* = .15; *p* = .1988	*r* = .31; *p* = .0072	*r* = .15; *p* = .2154	*r* = .33; *p* = .0041	*r* = .15; *p* = .2107	*r* = .30; *p* = .0090
	lBA10	*r* = .20; *p* = .0840	*r* = .29; *p* = .0138	*r* = .19; *p* = .1096	*r* = .35; *p* = .0026	*r* = .18; *p* = .1364	*r* = .27; *p* = .0198
	rBA10	*r* = .19; *p* = .1026	*r* = .19; *p* = .0991	*r* = .19; *p* = .1118	*r* = .27; *p* = .0206	*r* = .15; *p* = .2084	*r* = .22; *p* = .0650
	lBA11	*r* = .22; *p* = .0561	*r* = .36; *p* = .0015	*r* = .19; *p* = .0997	***r*** **= .44;** ***p*** **= .0001**	*r* = .23; *p* = .0462	*r* = .36; *p* = .0014
	rBA11	*r* = .14; *p* = .2434	*r* = .34; *p* = .0033	*r* = .13; *p* = .2546	***r*** **= .41;** ***p*** **= .0003**	*r* = .12; *p* = .3127	*r* = .38; *p* = .0009
	lBA46	*r* = .26; *p* = .0238	*r* = .40; *p* = .0005	*r* = .22; *p* = .0565	*r* = .36; *p* = .0015	*r* = .25; *p* = .0329	*r* = .36; *p* = .0014
	rBA46	*r* = .06; *p* = .6360	*r* = .20; *p* = .0908	*r* = .04; *p* = .7513	*r* = .20; *p* = .0906	*r* = .03; *p* = .7849	*r* = .19; *p* = .0978
	lBA47	*r* = .23; *p* = .0488	*r* = .32; *p* = .0049	*r* = .21; *p* = .0644	*r* = .37; *p* = .0013	*r* = .21; *p* = .0798	*r* = .29; *p* = .0112
	rBA47	*r* = −.01; *p* = .9092	*r* = .35; *p* = .0026	*r* = −.03; *p* = .7951	*r* = .36; *p* = .0015	*r* = −.03; *p* = .8304	*r* = .37; *p* = .0014

*Note.* ACC = anterior cingulate cortex; PCC = posterior cingulate cortex; PFC = prefrontal cortex

*p* value ≤.0003

For each BA that showed a significant correlation with the ECR-R scores, a multiple factorial regression analysis was performed using age, sex, anxiety, avoidance, Sex × Anxiety, And Sex × Avoidance as predictors. As reported in Table 5, the regression models were all significant. The avoidance predictor was always positively and statistically significant coherently with the inclusion criteria applied starting from the significant correlations. The sex predictor was significant (with women showing increased brain intensity compared with men) for the early and late prefrontal cortex ROI intensity in response to positive condition; for the early and late cingulate and prefrontal cortices ROIs intensity in response to negative condition; and for the early posterior cingulate cortex ROI intensity in response to ambiguous condition. Sex × Avoidance was a significant predictor for posterior cingulate and prefrontal cortices ROIs intensity in response to positive condition, and for early and late cingulate and prefrontal cortices ROIs intensity in response to the ambiguous and negative conditions.

**Table 5:** Multiple factorial regression analyses with age, sex, ECR-R-anxiety score, ECR-R-avoidance score, and the Sex × ECR-R Score interaction as predictors performed on the intensity of the left (l) and right (r) Brodmann Areas (BAs). The regression model was performed on those BAs for which the correlation (Pearson’s *r*) between the ECR-R scores (anxiety and avoidance) and the mean intensity of the left and right BA of each region of interest (ROI) [limbic ROI; anterior cingulate cortex ROI (ACC); posterior cingulate cortex ROI (PCC); and prefrontal cortex ROI (PFC)] was significant (*n* = 74)

Condition	ERP	ROI	BA	Regression model	Predictors					
					Age	Sex	Anxiety	Sex × Anxiety	Avoidance	Sex × Avoidance
Positive	P100	Limbic	rAMG	***R*** **= .46;** ***R***^**2**^ **= .21;** ***R***^**2**^_**adj**_ **= .14** ***F*(6, 67) = 3.02;** ***p*** **= .011;** ***SE*** **= 4.34**	β = .03; *SE* = .11; *t*(67) = .28; *p* = .783	β = −.10; *SE* = .11; *t*(67) = −.94; *p* = .350	β = .17; *SE* = .12; *t*(67) = 1.35; *p* = .181	β = .15; *SE* = .12; *t*(67) = 1.28; *p* = .203	**β = .39;** ***SE*** **= .11;** ***t*(67) = 3.41;** ***p*** **= .001**	β = −.12; *SE* = .11; *t*(67) = −1.06; *p* = .292
			rAHj	***R*** **= .45;** ***R***^**2**^ **= .21;** ***R***^**2**^_**adj**_ **= .14** ***F*(6, 67) = 2.97;** ***p*** **= .012;** ***SE*** **= 4.08**	β = .02; *SE* = .11; *t*(67) = .14; *p* = .886	β = −.11; *SE* = .11; *t*(67) = −.98; *p* = .332	β = .17; *SE* = .12; *t*(67) = 1.35; *p* = .180	β = .15; *SE* = .12; *t*(67) = 1.25; *p* = .214	**β = .39;** ***SE*** **= .11;** ***t*(67) = 3.38;** ***p*** **= .001**	β = −.11; *SE* = .11; *t*(67) = −.98; *p* = .327
			rHPC	***R*** **= .48;** ***R***^**2**^ **= .23;** ***R***^**2**^_**adj**_ **= .16** ***F*(6, 67) = 3.38;** ***p*** **= .006;** ***SE*** **= 4.14**	β = .07; *SE* = .11; *t*(67) = .62; *p* = .535	β = −.13; *SE* = .11; *t*(67) = −1.26; *p* = .212	β = .22; *SE* = .12; *t*(67) = 1.84; *p* = .070	β = .13; *SE* = .12; *t*(67) = 1.08; *p* = .284	**β = .38;** ***SE*** **= .11;** ***t*(67) = 3.35;** ***p*** **= .001**	β = −.09; *SE* = .11; *t*(67) = −.81; *p* = .418
	N200	Limbic	rAMG	***R*** **= .50;** ***R***^**2**^ **= .25;** ***R***^**2**^_**adj**_ **= .18** ***F*(6, 67) = 3.67;** ***p*** **= .003;** ***SE*** **= 4.45**	β = .08; *SE* = .11; *t*(67) = .77; *p* = .444	β = −.14; *SE* = .11; *t*(67) = −1.33; *p* = .187	β = .16; *SE* = .12; *t*(67) = 1.36; *p* = .179	β = .14; *SE* = .12; *t*(67) = 1.21; *p* = .230	**β = .42;** ***SE*** **= .11;** ***t*(67) = 3.75;** ***p*** **< .001**	β = −.14; *SE* = .11; *t*(67) = −1.29; *p* = .199
			rAHj	***R*** **= .50;** ***R***^**2**^ **= .25;** ***R***^**2**^_**adj**_ **= .18** ***F*(6, 67) = 3.69;** ***p*** **= .003;** ***SE*** **= 4.21**	β = .06; *SE* = .11; *t*(67) = .60; *p* = .553	β = −.15; *SE* = .11; *t*(67) = −1.36; *p* = .178	β = .16; *SE* = .12; *t*(67) = 1.35; *p* = .182	β = .15; *SE* = .12; *t*(67) = 1.23; *p* = .222	**β = .43;** ***SE*** **= .11;** ***t*(67) = 3.80;** ***p*** **< .001**	β = −.14; *SE* = .11; *t*(67) = −1.27; *p* = .207
		PCC	rBA23	***R*** **= .51;** ***R***^**2**^ **= .26;** ***R***^**2**^_**adj**_ **= .20** ***F*(6, 67) = 4.03;** ***p*** **= .002;** ***SE*** **= 1.91**	β = .09; *SE* = .11; *t*(67) = .88; *p* = .381	β = −20; *SE* = .11; *t*(67) = −1.85; *p* = .068	β = .12; *SE* = .12; *t*(67) = 1.04; *p* = .303	β = .14; *SE* = .12; *t*(67) = 1.22; *p* = .227	**β = .42;** ***SE*** **= .11;** ***t*(67) = 3.82;** ***p*** **< .001**	**β = −.23;** ***SE*** **= .11;** ***t*(67) = −2.15;** ***p*** **= .035**
		PFC	lBA11	***R*** **= .53;** ***R***^**2**^ **= .22;** ***R***^**2**^_**adj**_ **= .20** ***F*(6, 67) = 4.49;** ***p*** **< .001;** ***SE*** **= 3.18**	β = .01; *SE* = .10; *t*(67) = .09; *p* = .926	**β = −.21;** ***SE*** **= .10;** ***t*(67) = −2.02;** ***p*** **= .047**	β = .10; *SE* = .12; *t*(67) = .89; *p* = .377	β = .10; *SE* = .12; *t*(67) = .89; *p* = .374	**β = .45;** ***SE*** **= .11;** ***t*(67) = 4.11;** ***p*** **< .001**	**β = −.26;** ***SE*** **= .11;** ***t*(67) = −2.44;** ***p*** **= .017**
	P250	Limbic	rAMG	***R*** **= .50;** ***R***^**2**^ **= .25;** ***R***^**2**^_**adj**_ **= .18** ***F*(6, 67) = 3.77;** ***p*** **= .003;** ***SE*** **= 4.44**	β = .09; *SE* = .11; *t*(67) = .81; *p* = .419	β = −.16; *SE* = .11; *t*(67) = −1.51; *p* = .135	β = .16; *SE* = .12; *t*(67) = 1.31; *p* = .193	β = .14; *SE* = .12; *t*(67) = 1.21; *p* = .229	**β = .42;** ***SE*** **= .11;** ***t*(67) = 3.77;** ***p*** **< .001**	β = −.16; *SE* = .11; *t*(67) = −1.49; *p* = .140
			rAHj	***R*** **= .50;** ***R***^**2**^ **= .25;** ***R***^**2**^_**adj**_ **= .18** ***F*(6, 67) = 3.73;** ***p*** **= .003;** ***SE*** **= 4.14**	β = .05; *SE* = .11; *t*(67) = .44; *p* = .662	β = −.14; *SE* = .11; *t*(67) = −1.37; *p* = .175	β = .15; *SE* = .12; *t*(67) = 1.45; *p* = .196	β = .17; *SE* = .12; *t*(67) = 1.45; *p* = .153	**β = .43;** ***SE*** **= .11;** ***t*(67) = 3.79;** ***p*** **< .001**	β = −.17; *SE* = .11; *t*(67) = −1.53; *p* = .129
			rHPC	***R*** **= .52;** ***R***^**2**^ **= .27;** ***R***^**2**^_**adj**_ **= .20** ***F*(6, 67) = 4.09;** ***p*** **= .001;** ***SE*** **= 4.26**	β = .10; *SE* = .11; *t*(67) = .98; *p* = .330	β = −.20; *SE* = .11; *t*(67) = −1.85; *p* = .068	β = .19; *SE* = .12; *t*(67) = 1.65; *p* = .103	β = .10; *SE* = .12; *t*(67) = .83; *p* = .408	**β = .41;** ***SE*** **= .11;** ***t*(67) = 3.68;** ***p*** **< .001**	β = −.17; *SE* = .11; *t*(67) = −1.59; *p* = .116
		PFC	lBA11	***R*** **= .57;** ***R***^**2**^ **= .32;** ***R***^**2**^_**adj**_ **= .26** ***F*(6, 67) = 5.36;** ***p*** **< .001;** ***SE*** **= 3.16**	β = −.02; *SE* = .10; *t*(67) = −.17; *p* = .864	**β = −.21;** ***SE*** **= .10;** ***t*(67) = −2.02;** ***p*** **= .047**	β = .10; *SE* = .11; *t*(67) = .89; *p* = .376	β = .10; *SE* = .11; *t*(67) = .89; *p* = .375	**β = .47;** ***SE*** **= .11;** ***t*(67) = 4.37;** ***p*** **< .001**	**β = −.32;** ***SE*** **= .10;** ***t*(67) = −3.04;** ***p*** **= .003**
	LC1	Limbic	rAMG	***R*** **= .48;** ***R***^**2**^ **= .23;** ***R***^**2**^_**adj**_ **= .17** ***F*(6, 67) = 3.45;** ***p*** **= .005;** ***SE*** **= 4.30**	β = .04; *SE* = .11; *t*(67) = .41; *p* = .679	β = −.13; *SE* = .10; *t*(67) = −1.23; *p* = .221	β = .17; *SE* = .12; *t*(67) = 1.39; *p* = .170	β = .13; *SE* = .12; *t*(67) = 1.07; *p* = .289	**β = .41;** ***SE*** **= .11;** ***t*(67) = 3.65;** ***p*** **< .001**	β = −.14; *SE* = .11; *t*(67) = −1.24; *p* = .216
			rAHj	***R*** **= .48;** ***R***^**2**^ **= .23;** ***R***^**2**^_**adj**_ **= .17** ***F*(6, 67) = 3.42;** ***p*** **= .005;** ***SE*** **= 4.04**	β = .01; *SE* = .11; *t*(67) = .14; *p* = .887	β = −.12; *SE* = .11; *t*(67) = −1.10; *p* = .276	β = .17; *SE* = .12; *t*(67) = 1.46; *p* = .149	β = .14; *SE* = .12; *t*(67) = 1.15; *p* = .253	**β = .41;** ***SE*** **= .11;** ***t*(67) = 3.62;** ***p*** **< .001**	β = −.13; *SE* = .11; *t*(67) = −1.20; *p* = .234
			rHPC	***R*** **= .50;** ***R***^**2**^ **= .25;** ***R***^**2**^_**adj**_ **= .18** ***F*(6, 67) = 3.72;** ***p*** **= .003;** ***SE*** **= 4.18**	β = .08; *SE* = .11; *t*(67) = .70; *p* = .483	β = −.17; *SE* = .11; *t*(67) = −1.55; *p* = .127	β = .22; *SE* = .12; *t*(67) = 1.83; *p* = .071	β = .10; *SE* = .12; *t*(67) = .83; *p* = .406	**β = .39;** ***SE*** **= .11;** ***t*(67) = 3.50;** ***p*** **= .001**	β = −.13; *SE* = .11; *t*(67) = −1.15; *p* = .253
	LC2	Limbic	rAMG	***R*** **= .49;** ***R***^**2**^ **= .24;** ***R***^**2**^_**adj**_ **= .17** ***F*(6, 67) = 3.55;** ***p*** **= .004;** ***SE*** **= 4.19**	β = .07; *SE* = .11; *t*(67) = .61; *p* = .541	β = −.14; *SE* = .11; *t*(67) = −1.26; *p* = .211	β = .16; *SE* = .12; *t*(67) = 1.37; *p* = .176	β = .12; *SE* = .12; *t*(67) = 1.06; *p* = .294	**β = .42;** ***SE*** **= .11;** ***t*(67) = 3.71;** ***p*** **< .001**	β = −.14; *SE* = .11; *t*(67) = −1.29; *p* = .201
			rAHj	***R*** **= .49;** ***R***^**2**^ **= .24;** ***R***^**2**^_**adj**_ **= .17** ***F*(6, 67) = 3.54;** ***p*** **= .004;** ***SE*** **= 3.90**	β = .03; *SE* = .11; *t*(67) = .32; *p* = .749	β = −.12; *SE* = .11; *t*(67) = −1.14; *p* = .259	β = .17; *SE* = .12; *t*(67) = 1.42; *p* = .159	β = .14; *SE* = .12; *t*(67) = 1.15; *p* = .252	**β = .42;** ***SE*** **= .11;** ***t*(67) = 3.70;** ***p*** **< .001**	β = −.15; *SE* = .11; *t*(67) = −1.35; *p* = .183
			rHPC	***R*** **= .50;** ***R***^**2**^ **= .25;** ***R***^**2**^_**adj**_ **= .19** ***F*(6, 67) = 3.77;** ***p*** **= .003;** ***SE*** **= 4.15**	β = .10; *SE* = .11; *t*(67) = .91; *p* = .365	β = −.17; *SE* = .11; *t*(67) = −1.57; *p* = .124	β = .23; *SE* = .12; *t*(67) = 1.93; *p* = .058	β = .23; *SE* = .12; *t*(67) = .77; *p* = .441	**β = .39;** ***SE*** **= .11;** ***t*(67) = 3.44;** ***p*** **= .001**	β = −.12; *SE* = .11; *t*(67) = −1.10; *p* = .275
Negative	P100	Limbic	rAMG	***R*** **= .46;** ***R***^**2**^ **= .22;** ***R***^**2**^_**adj**_ **= .15** ***F*(6, 67) = 3.08;** ***p*** **= .010;** ***SE*** **= 4.49**	β = .03; *SE* = .11; *t*(67) = .25; *p* = .805	β = −.14; *SE* = .11; *t*(67) = −1.30; *p* = .196	β = .15; *SE* = .12; *t*(67) = 1.26; *p* = .210	β = .09; *SE* = .12; *t*(67) = .77; *p* = .443	**β = .40;** ***SE*** **= .11;** ***t*(67) = 3.50;** ***p*** **= .001**	β = −.12; *SE* = .11; *t*(67) = −1.04; *p* = .302
			rAHj	***R*** **= .46;** ***R***^**2**^ **= .22;** ***R***^**2**^_**adj**_ **= .14** ***F*(6, 67) = 3.07;** ***p*** **= .010;** ***SE*** **= 4.18**	β = .003; *SE* = .11; *t*(67) = .03; *p* = .976	β = −.13; *SE* = .11; *t*(67) = −1.13; *p* = .260	β = .15; *SE* = .12; *t*(67) = 1.21; *p* = .231	β = .11; *SE* = .12; *t*(67) = .92; *p* = .360	**β = .41;** ***SE*** **= .11;** ***t*(67) = 3.55;** ***p*** **= .001**	β = −.11; *SE* = .11; *t*(67) = −.99; *p* = .323
		ACC	lBA32	***R*** **= .54;** ***R***^**2**^ **= .30;** ***R***^**2**^_**adj**_ **= .23** ***F*(6, 67) = 4.72;** ***p*** **< .001;** ***SE*** **= 1.26**	β = −.09; *SE* = .10; *t*(67) = −.85; *p* = .400	β = −.19; *SE* = .10; *t*(67) = −1.78; *p* = .078	β = .12; *SE* = .11; *t*(67) = 1.08; *p* = .282	β = .14; *SE* = .11; *t*(67) = 1.27; *p* = .209	**β = .45;** ***SE*** **= .11;** ***t*(67) = 4.22;** ***p*** **< .001**	**β = −.24;** ***SE*** **= .11;** ***t*(67) = −2.23;** ***p*** **= .029**
			rBA32	***R*** **= .47;** ***R***^**2**^ **= .22;** ***R***^**2**^_**adj**_ **= .15** ***F*(6, 67) = 3.22;** ***p*** **= .008;** ***SE*** **= 1.01**	β = −.09; *SE* = .11; *t*(67) = −.79; *p* = .433	β = −.07; *SE* = .11; *t*(67) = −.68; *p* = .500	β = .14; *SE* = .12; *t*(67) = 1.20; *p* = .232	β = .18; *SE* = .12; *t*(67) = 1.48; *p* = .143	**β = .40;** ***SE*** **= .11;** ***t*(67) = 3.53;** ***p*** **< .001**	β = −.12; *SE* = .11; *t*(67) = −1.07; *p* = .287
		PCC	rBA23	***R*** **= .49;** ***R***^**2**^ **= .23;** ***R***^**2**^_**adj**_ **= .17** ***F*(6, 67) = 3.45;** ***p*** **= .005;** ***SE*** **= 1.90**	β = .07; *SE* = .11; *t*(67) = .67; *p* = .502	β = −.18; *SE* = .11; *t*(67) = −1.64; *p* = .106	β = .08; *SE* = .12; *t*(67) = .66; *p* = .512	β = .10; *SE* = .12; *t*(67) = .88; *p* = .380	**β = .43;** ***SE*** **= .11;** ***t*(67) = 3.82;** ***p*** **< .001**	β = −.18; *SE* = .11; *t*(67) = −.44; *p* = .105
		PFC	lBA11	***R*** **= .52;** ***R***^**2**^ **= .27;** ***R***^**2**^_**adj**_ **= .20** ***F*(6, 67) = 4.13;** ***p*** **= .001;** ***SE*** **= 3.10**	β = −.01; *SE* = .11; *t*(67) = −.14; *p* = .886	β = −.15; *SE* = .11; *t*(67) = −1.38; *p* = .170	β = .08; *SE* = .12; *t*(67) = .69; *p* = .493	β = .14; *SE* = .12; *t*(67) = 1.18; *p* = .243	**β = .46;** ***SE*** **= .11;** ***t*(67) = 4.13;** ***p*** **< .001**	**β = −.25;** ***SE*** **= .11;** ***t*(67) = −2.32;** ***p*** **= .023**
	N200	Limbic	rAMG	***R*** **= .50;** ***R***^**2**^ **= .25;** ***R***^**2**^_**adj**_ **= .18** ***F*(6, 67) = 3.66;** ***p*** **= .003;** ***SE*** **= 4.48**	β = .02; *SE* = .11; *t*(67) = .21; *p* = .831	β = −15; *SE* = .11; *t*(67) = −1.42; *p* = .159	β = .10; *SE* = .12; *t*(67) = .83; *p* = .412	β = .12; *SE* = .12; *t*(67) = 1.00; *p* = .321	**β = .45;** ***SE*** **= .11;** ***t*(67) = 3.99;** ***p*** **< .001**	β = −.18; *SE* = .11; *t*(67) = −1.64; *p* = .105
			rAHj	***R*** **= .50;** ***R***^**2**^ **= .25;** ***R***^**2**^_**adj**_ **= .18** ***F*(6, 67) = 3.65;** ***p*** **= .003;** ***SE*** **= 4.26**	β = .01; *SE* = .11; *t*(67) = .11; *p* = .913	β = −15; *SE* = .11; *t*(67) = −1.38; *p* = .172	β = .10; *SE* = .12; *t*(67) = .97; *p* = .336	β = .11; *SE* = .12; *t*(67) = .97; *p* = .336	**β = .45;** ***SE*** **= .11;** ***t*(67) = 4.00;** ***p*** **< .001**	β = −.17; *SE* = .11; *t*(67) = −1.58; *p* = .118
			rHPC	***R*** **= .48;** ***R***^**2**^ **= .23;** ***R***^**2**^_**adj**_ **= .16** ***F*(6, 67) = 3.35;** ***p*** **= .006;** ***SE*** **= 4.24**	β = .04; *SE* = .11; *t*(67) = .36; *p* = .718	β = −17; *SE* = .11; *t*(67) = −1.55; *p* = .125	β = .14; *SE* = .12; *t*(67) = 1.12; *p* = .264	β = .05; *SE* = .12; *t*(67) = .42; *p* = .670	**β = .41;** ***SE*** **= .11;** ***t*(67) = 3.63;** ***p*** **< .001**	β = −.16; *SE* = .11; *t*(67) = −1.39; *p* = .167
		ACC	lBA32	***R*** **= .57;** ***R***^**2**^ **= .33;** ***R***^**2**^_**adj**_ **= .27** ***F*(6, 67) = 5.50;** ***p*** **< .001;** ***SE*** **= 1.28**	β = −.11; *SE* = .11; *t*(67) = −1.09; *p* = .279	**β = −21;** ***SE*** **= .10;** ***t*(67) = −2.02;** ***p*** **= .047**	β = .07; *SE* = .11; *t*(67) = .58; *p* = .561	β = .15; *SE* = .11; *t*(67) = 1.32; *p* = .190	**β = .48;** ***SE*** **= .11;** ***t*(67) = 4.54;** ***p*** **< .001**	**β = −.30;** ***SE*** **= .10;** ***t*(67) = −2.89;** ***p*** **= .005**
			rBA32	***R*** **= .51;** ***R***^**2**^ **= .27;** ***R***^**2**^_**adj**_ **= .20** ***F*(6, 67) = 4.13;** ***p*** **= .001;** ***SE*** **= 1.08**	β = −.08; *SE* = .11; *t*(67) = −.77; *p* = .440	β = −11; *SE* = .11; *t*(67) = −1.06; *p* = .294	β = .09; *SE* = .12; *t*(67) = .79; *p* = .433	β = .18; *SE* = .12; *t*(67) = 1.54; *p* = .128	**β = .46;** ***SE*** **= .11;** ***t*(67) = 4.15;** ***p*** **< .001**	β = −.22; *SE* = .11; *t*(67) = −2.00; *p* = .050
			rBA33	***R*** **= .48;** ***R***^**2**^ **= .23;** ***R***^**2**^_**adj**_ **= .16** ***F*(6, 67) = 3.30;** ***p*** **= .007;** ***SE*** **= 1.47**	β = −.03; *SE* = .11; *t*(67) = −.25; *p* = .807	β = −11; *SE* = .11; *t*(67) = −1.02; *p* = .312	β = .10; *SE* = .12; *t*(67) = .87; *p* = .387	β = .17; *SE* = .12; *t*(67) = 1.44; *p* = .153	**β = .42;** ***SE*** **= .11;** ***t*(67) = 3.68;** ***p*** **< .001**	β = −.19; *SE* = .11; *t*(67) = −1.73; *p* = .087
		PFC	lBA11	***R*** **= .55;** ***R***^**2**^ **= .30;** ***R***^**2**^_**adj**_ **= .24** ***F*(6, 67) = 4.82;** ***p*** **< .001;** ***SE*** **= 3.56**	β = −.01; *SE* = .11; *t*(67) = −.20; *p* = .843	β = −18; *SE* = .10; *t*(67) = −1.71; *p* = .091	β = .04; *SE* = .11; *t*(67) = .37; *p* = .714	β = .12; *SE* = .11; *t*(67) = 1.10; *p* = .275	**β = .48;** ***SE*** **= .11;** ***t*(67) = 4.41;** ***p*** **< .001**	**β = −.30;** ***SE*** **= .11;** ***t*(67) = −2.84;** ***p*** **= .006**
			rBA11	***R*** **= .48;** ***R***^**2**^ **= .23;** ***R***^**2**^_**adj**_ **= .16** ***F*(6, 67) = 3.39;** ***p*** **= .005;** ***SE*** **= 2.47**	β = −.05; *SE* = .11; *t*(67) = −.46; *p* = .649	β = −.09; *SE* = .11; *t*(67) = −.78; *p* = .437	β = .09; *SE* = .11; *t*(67) = .74; *p* = .462	β = .22; *SE* = .12; *t*(67) = 1.84; *p* = .070	**β = .42;** ***SE*** **= .11;** ***t*(67) = 3.71;** ***p*** **< .001**	β = −.19; *SE* = .11; *t*(67) = −1.70; *p* = .093
	P250	Limbic	rAMG	***R*** **= .49;** ***R***^**2**^ **= .24;** ***R***^**2**^_**adj**_ **= .17** ***F*(6, 67) = 3.53;** ***p*** **= .004;** ***SE*** **= 4.04**	β = .05; *SE* = .11; *t*(67) = .45; *p* = .650	β = −.18; *SE* = .11; *t*(67) = −1.69; *p* = .095	β = .12; *SE* = .12; *t*(67) = 1.04; *p* = .302	β = .09; *SE* = .12; *t*(67) = .73; *p* = .468	**β = .44;** ***SE*** **= .11;** ***t*(67) = 3.80;** ***p*** **< .001**	β = −.15; *SE* = .11; *t*(67) = −1.38; *p* = .172
			rAHj	***R*** **= .49;** ***R***^**2**^ **= .23;** ***R***^**2**^_**adj**_ **= .17** ***F*(6, 67) = 3.41;** ***p*** **= .005;** ***SE*** **= 3.88**	β = .19; *SE* = .11; *t*(67) = .17; *p* = .860	β = −.16; *SE* = .11; *t*(67) = −1.44; *p* = .152	β = .12; *SE* = .12; *t*(67) = .97; *p* = .333	β = .10; *SE* = .12; *t*(67) = .86; *p* = .392	**β = .43;** ***SE*** **= .11;** ***t*(67) = 3.81;** ***p*** **< .001**	β = −.15; *SE* = .11; *t*(67) = −.74; *p* = .182
		ACC	rBA24	***R*** **= .47;** ***R***^**2**^ **= .22;** ***R***^**2**^_**adj**_ **= .15** ***F*(6, 67) = 3.22;** ***p*** **= .008;** ***SE*** **= 1.46**	β = .05; *SE* = .11; *t*(67) = .46; *p* = .645	β = −.09; *SE* = .11; *t*(67) = −.84; *p* = .400	β = .11; *SE* = .12; *t*(67) = .89; *p* = .377	β = .21; *SE* = .12; *t*(67) = 1.77; *p* = .080	**β = .41;** ***SE*** **= .11;** ***t*(67) = 3.57;** ***p*** **< .001**	β = −.17; *SE* = .11; *t*(67) = −1.55; *p* = .123
			lBA32	***R*** **= .58;** ***R***^**2**^ **= .34;** ***R***^**2**^_**adj**_ **= .28** ***F*(6, 67) = 5.72;** ***p*** **< .001;** ***SE*** **= 1.18**	β = −.11; *SE* = .10; *t*(67) = −1.11; *p* = .269	**β = −.20;** ***SE*** **= .10;** ***t*(67) = −2.02;** ***p*** **= .046**	β = .09; *SE* = .11; *t*(67) = .82; *p* = .412	β = .14; *SE* = .11; *t*(67) = 1.24; *p* = .219	**β = .49;** ***SE*** **= .11;** ***t*(67) = 4.65;** ***p*** **< .001**	**β = −.28;** ***SE*** **= .10;** ***t*(67) = −2.72;** ***p*** **= .008**
			rBA32	***R*** **= .52;** ***R***^**2**^ **= .27;** ***R***^**2**^_**adj**_ **= .21** ***F*(6, 67) = 4.20;** ***p*** **= .001;** ***SE*** **= 0.94**	β = −.08; *SE* = .11; *t*(67) = −.80; *p* = .422	β = −.11; *SE* = .11; *t*(67) = −1.02; *p* = .311	β = .11; *SE* = .12; *t*(67) = .95; *p* = .344	β = .17; *SE* = .12; *t*(67) = 1.46; *p* = .148	**β = .46;** ***SE*** **= .11;** ***t*(67) = 4.17;** ***p*** **< .001**	β = −.20; *SE* = .11; *t*(67) = −1.83; *p* = .071
		PCC	rBA23	***R*** **= .52;** ***R***^**2**^ **= .28;** ***R***^**2**^_**adj**_ **= .21** ***F*(6, 67) = 4.25;** ***p*** **= .001;** ***SE*** **= 1.89**	β = .06; *SE* = .11; *t*(67) = .57; *p* = .571	β = −.20; *SE* = .11; *t*(67) = −1.91; *p* = .060	β = .06; *SE* = .12; *t*(67) = .51; *p* = .609	β = .11; *SE* = .12; *t*(67) = .95; *p* = .343	**β = .45;** ***SE*** **= .11;** ***t*(67) = 4.11;** ***p*** **< .001**	**β = −.26;** ***SE*** **= .10;** ***t*(67) = −2.41;** ***p*** **= .018**
		PFC	lBA11	***R*** **= .55;** ***R***^**2**^ **= .31;** ***R***^**2**^_**adj**_ **= .25** ***F*(6, 67) = 4.97;** ***p*** **< .001;** ***SE*** **= 2.94**	β = −.08; *SE* = .10; *t*(67) = −.73; *p* = .465	β = −.19; *SE* = .10; *t*(67) = −1.81; *p* = .075	β = .06; *SE* = .11; *t*(67) = .52; *p* = .607	β = .11; *SE* = .11; *t*(67) = .95; *p* = .343	**β = .48;** ***SE*** **= .11;** ***t*(67) = 4.47;** ***p*** **< .001**	**β = −.28;** ***SE*** **= .11;** ***t*(67) = −2.67;** ***p*** **= .010**
	LC1	Limbic	rAMG	***R*** **= .51;** ***R***^**2**^ **= .26;** ***R***^**2**^_**adj**_ **= .19** ***F*(6, 67) = 3.89;** ***p*** **= .002;** ***SE*** **= 4.30**	β = .06; *SE* = .11; *t*(67) = .58; *p* = .562	β = −.18; *SE* = .11; *t*(67) = −1.65; *p* = .103	β = .11; *SE* = .12; *t*(67) = .94; *p* = .350	β = .09; *SE* = .12; *t*(67) = .78; *p* = .440	**β = .46;** ***SE*** **= .11;** ***t*(67) = 4.09;** ***p*** **< .001**	β = −.15; *SE* = .11; *t*(67) = −1.40; *p* = .164
			rAHj	***R*** **= .51;** ***R***^**2**^ **= .26;** ***R***^**2**^_**adj**_ **= .19** ***F*(6, 67) = 3.94;** ***p*** **= .002;** ***SE*** **= 4.01**	β = .04; *SE* = .11; *t*(67) = .35; *p* = .730	β = −.16; *SE* = .11; *t*(67) = −1.52; *p* = .133	β = .11; *SE* = .12; *t*(67) = .91; *p* = .332	β = .11; *SE* = .12; *t*(67) = .91; *p* = .367	**β = .46;** ***SE*** **= .11;** ***t*(67) = 4.15;** ***p*** **< .001**	β = −.15; *SE* = .11; *t*(67) = −1.38; *p* = .173
			rHPC	***R*** **= .51;** ***R***^**2**^ **= .26;** ***R***^**2**^_**adj**_ **= .19** ***F*(6, 67) = 3.86;** ***p*** **= .002;** ***SE*** **= 4.23**	β = .07; *SE* = .11; *t*(67) = .68; *p* = .496	β = −.20; *SE* = .11; *t*(67) = −1.86; *p* = .067	β = .16; *SE* = .12; *t*(67) = 1.40; *p* = .166	β = .05; *SE* = .12; *t*(67) = .44; *p* = .661	**β = .42;** ***SE*** **= .11;** ***t*(67) = 3.76;** ***p*** **< .001**	β = −.14; *SE* = .11; *t*(67) = −1.28; *p* = .203
		ACC	lBA32	***R*** **= .59;** ***R***^**2**^ **= .34;** ***R***^**2**^_**adj**_ **= .28** ***F*(6, 67) = 5.84;** ***p*** **< .001;** ***SE*** **= 1.27**	β = −.10; *SE* = .10; *t*(67) = −.98; *p* = .327	**β = −.21;** ***SE*** **= .10;** ***t*(67) = −2.08;** ***p*** **= .041**	β = .10; *SE* = .11; *t*(67) = .90; *p* = .371	β = .14; *SE* = .11; *t*(67) = 1.29; *p* = .202	**β = .49;** ***SE*** **= .10;** ***t*(67) = 4.67;** ***p*** **< .001**	**β = −.29;** ***SE*** **= .10;** ***t*(67) = −2.81;** ***p*** **= .006**
			rBA32	***R*** **= .52;** ***R***^**2**^ **= .27;** ***R***^**2**^_**adj**_ **= .21** ***F*(6, 67) = 4.21;** ***p*** **< .001;** ***SE*** **= 0.94**	β = −.07; *SE* = .11; *t*(67) = −.62; *p* = .535	β = −.14; *SE* = .11; *t*(67) = −1.30; *p* = .197	β = .14; *SE* = .12; *t*(67) = 1.17; *p* = .245	β = .17; *SE* = .12; *t*(67) = 1.48; *p* = .143	**β = .44;** ***SE*** **= .11;** ***t*(67) = 4.02;** ***p*** **< .001**	**β = −.22;** ***SE*** **= .11;** ***t*(67) = −2.03;** ***p*** **= .046**
			lBA33	***R*** **= .49;** ***R***^**2**^ **= .24;** ***R***^**2**^_**adj**_ **= .18** ***F*(6, 67) = 3.59;** ***p*** **= .004;** ***SE*** **= 1.40**	β = −.05; *SE* = .11; *t*(67) = −.54; *p* = .591	β = −.15; *SE* = .11; *t*(67) = −1.36; *p* = .177	β = .13; *SE* = .12; *t*(67) = 1.14; *p* = .272	β = .13; *SE* = .12; *t*(67) = 1.14; *p* = .257	**β = .42;** ***SE*** **= .11;** ***t*(67) = 3.72;** ***p*** **< .001**	β = −.20; *SE* = .11; *t*(67) = −1.82; *p* = .073
			rBA33	***R*** **= .48;** ***R***^**2**^ **= .23;** ***R***^**2**^_**adj**_ **= .16** ***F*(6, 67) = 3.34;** ***p*** **= .006;** ***SE*** **= 1.34**	β = −.02; *SE* = .11; *t*(67) = −.20; *p* = .842	β = −.13; *SE* = .11; *t*(67) = −1.18; *p* = .242	β = .15; *SE* = .12; *t*(67) = 1.22; *p* = .228	β = .15; *SE* = .12; *t*(67) = 1.27; *p* = .210	**β = .41;** ***SE*** **= .11;** ***t*(67) = 3.59;** ***p*** **< .001**	β = −.18; *SE* = .11; *t*(67) = −1.63; *p* = .106
		PCC	lBA23	***R*** **= .51;** ***R***^**2**^ **= .26;** ***R***^**2**^_**adj**_ **= .20** ***F*(6, 67) = 4.02;** ***p*** **= .002;** ***SE*** **= 1.91**	β = .17; *SE* = .11; *t*(67) = 1.61; *p* = .112	β = −.20; *SE* = .11; *t*(67) = −1.83; *p* = .070	β = .04; *SE* = .12; *t*(67) = .33; *p* = .743	β = .09; *SE* = .12; *t*(67) = .74; *p* = .459	**β = .44;** ***SE*** **= .11;** ***t*(67) = 3.98;** ***p*** **< .001**	β = −.20; *SE* = .11; *t*(67) = −1.87; *p* = .065
			rBA23	***R*** **= .54;** ***R***^**2**^ **= .29;** ***R***^**2**^_**adj**_ **= .23** ***F*(6, 67) = 4.66;** ***p*** **< .001;** ***SE*** **= 1.91**	β = .11; *SE* = .10; *t*(67) = 1.09; *p* = .280	**β = −.21;** ***SE*** **= .10;** ***t*(67) = −4.29;** ***p*** **= .047**	β = .06; *SE* = .11; *t*(67) = .51; *p* = .610	β = .10; *SE* = .11; *t*(67) = .91; *p* = .365	**β = .47;** ***SE*** **= .11;** ***t*(67) = 4.29;** ***p*** **< .001**	**β = −.25;** ***SE*** **= .11;** ***t*(67) = −2.37;** ***p*** **= .020**
			rBA30	***R*** **= .49;** ***R***^**2**^ **= .24;** ***R***^**2**^_**adj**_ **= .17** ***F*(6, 67) = 3.45;** ***p*** **= .005;** ***SE*** **= 3.04**	β = .05; *SE* = .11; *t*(67) = .49; *p* = .625	β = −.18; *SE* = .11; *t*(67) = −1.64; *p* = .106	β = .13; *SE* = .12; *t*(67) = 1.09; *p* = .279	β = .05; *SE* = .12; *t*(67) = .41; *p* = .679	**β = .42;** ***SE*** **= .11;** ***t*(67) = 3.67;** ***p*** **< .001**	β = −.16; *SE* = .11; *t*(67) = −1.47; *p* = .147
			rBA31	***R*** **= .60;** ***R***^**2**^ **= .36;** ***R***^**2**^_**adj**_ **= .30** ***F*(6, 67) = 6.22;** ***p*** **< .001;** ***SE*** **= 1.65**	β = .18; *SE* = .10; *t*(67) = 1.85; *p* = .068	**β = −.26;** ***SE*** **= .10;** ***t*(67) = −2.62;** ***p*** **= .011**	β = .01; *SE* = .11; *t*(67) = .05; *p* = .962	β = .15; *SE* = .11; *t*(67) = 1.40; *p* = .166	**β = .47;** ***SE*** **= .10;** ***t*(67) = 4.56;** ***p*** **< .001**	**β = −.33;** ***SE*** **= .10;** ***t*(67) = −3.19;** ***p*** **= .002**
		PFC	lBA11	***R*** **= .59;** ***R***^**2**^ **= .35;** ***R***^**2**^_**adj**_ **= .29** ***F*(6, 67) = 6.09;** ***p*** **< .001;** ***SE*** **= 3.18**	β = −.02; *SE* = .10; *t*(67) = −.22; *p* = .824	**β = −.21;** ***SE*** **= .10;** ***t*(67) = −2.22;** ***p*** **= .030**	β = .04; *SE* = .11; *t*(67) = .33; *p* = .738	β = .14; *SE* = .11; *t*(67) = 1.31; *p* = .738	**β = .51;** ***SE*** **= .10;** ***t*(67) = 4.88;** ***p*** **< .001**	**β = −.33;** ***SE*** **= .10;** ***t*(67) = −3.23;** ***p*** **= .002**
			rBA11	***R*** **= .48;** ***R***^**2**^ **= .23;** ***R***^**2**^_**adj**_ **= .17** ***F*(6, 67) = 3.42;** ***p*** **= .005;** ***SE*** **= 2.31**	β = −.01; *SE* = .11; *t*(67) = −.12; *p* = .906	β = −.10; *SE* = .11; *t*(67) = −.94; *p* = .351	β = .07; *SE* = .12; *t*(67) = .57; *p* = .568	β = .22; *SE* = .12; *t*(67) = 1.87; *p* = .066	**β = .43;** ***SE*** **= .11;** ***t*(67) = 3.78;** ***p*** **< .001**	β = −.19; *SE* = .11; *t*(67) = −1.72; *p* = .090
	LC2	Limbic	rAMG	***R*** **= .51;** ***R***^**2**^ **= .26;** ***R***^**2**^_**adj**_ **= .19** ***F*(6, 67) = 3.92;** ***p*** **= .002;** ***SE*** **= 4.31**	β = .06; *SE* = .11; *t*(67) = .58; *p* = .566	β = −.18; *SE* = .11; *t*(67) = −1.63; *p* = .107	β = .15; *SE* = .12; *t*(67) = 1.26; *p* = .212	β = .08; *SE* = .12; *t*(67) = .71; *p* = .477	**β = .44;** ***SE*** **= .11;** ***t*(67) = 3.94;** ***p*** **< .001**	β = −.15; *SE* = .11; *t*(67) = −1.39; *p* = .170
			rAHj	***R*** **= .51;** ***R***^**2**^ **= .26;** ***R***^**2**^_**adj**_ **= .19** ***F*(6, 67) = 3.91;** ***p*** **= .002;** ***SE*** **= 3.99**	β = .03; *SE* = .11; *t*(67) = .31; *p* = .758	β = −.15; *SE* = .11; *t*(67) = −1.41; *p* = .164	β = .15; *SE* = .12; *t*(67) = 1.27; *p* = .209	β = .10; *SE* = .12; *t*(67) = .82; *p* = .417	**β = .45;** ***SE*** **= .11;** ***t*(67) = 4.00;** ***p*** **< .001**	β = −.15; *SE* = .11; *t*(67) = −1.36; *p* = .179
			rHPC	***R*** **= .51;** ***R***^**2**^ **= .26;** ***R***^**2**^_**adj**_ **= .19** ***F*(6, 67) = 3.91;** ***p*** **= .002;** ***SE*** **= 4.27**	β = .07; *SE* = .11; *t*(67) = .66; *p* = .512	β = −.21; *SE* = .11; *t*(67) = −1.91; *p* = .060	β = .18; *SE* = .12; *t*(67) = 1.55; *p* = .125	β = .03; *SE* = .12; *t*(67) = .27; *p* = .783	**β = .41;** ***SE*** **= .11;** ***t*(67) = 3.65;** ***p*** **< .001**	β = −.15; *SE* = .11; *t*(67) = −1.33; *p* = .187
		ACC	lBA32	***R*** **= .58;** ***R***^**2**^ **= .34;** ***R***^**2**^_**adj**_ **= .28** ***F*(6, 67) = 5.67;** ***p*** **< .001;** ***SE*** **= 1.27**	β = −.10; *SE* = .10; *t*(67) = −1.02; *p* = .313	**β = −.21;** ***SE*** **= .10;** ***t*(67) = −2.02;** ***p*** **= .048**	β = .12; *SE* = .11; *t*(67) = 1.06; *p* = .291	β = .13; *SE* = .11; *t*(67) = 1.16; *p* = .250	**β = .47;** ***SE*** **= .11;** ***t*(67) = 4.47;** ***p*** **< .001**	**β = −.30;** ***SE*** **= .10;** ***t*(67) = −2.86;** ***p*** **= .006**
			rBA32	***R*** **= .53;** ***R***^**2**^ **= .29;** ***R***^**2**^_**adj**_ **= .22** ***F*(6, 67) = 4.48;** ***p*** **< .001;** ***SE*** **= 0.87**	β = −.06; *SE* = .10; *t*(67) = −.59; *p* = .557	β = −.13; *SE* = .11; *t*(67) = −1.27; *p* = .206	β = .15; *SE* = .12; *t*(67) = 1.28; *p* = .205	β = .17; *SE* = .11; *t*(67) = 1.48; *p* = .143	**β = .44;** ***SE*** **= .11;** ***t*(67) = 4.02;** ***p*** **< .001**	**β = −.26;** ***SE*** **= .11;** ***t*(67) = −2.40;** ***p*** **= .019**
			lBA33	***R*** **= .52;** ***R***^**2**^ **= .27;** ***R***^**2**^_**adj**_ **= .20** ***F*(6, 67) = 4.10;** ***p*** **= .001;** ***SE*** **= 1.34**	β = −.06; *SE* = .11; *t*(67) = −.57; *p* = .572	β = −.15; *SE* = .11; *t*(67) = −1.47; *p* = .146	β = .15; *SE* = .12; *t*(67) = 1.29; *p* = .201	β = .13; *SE* = .12; *t*(67) = 1.09; *p* = .281	**β = .42;** ***SE*** **= .11;** ***t*(67) = 3.81;** ***p*** **< .001**	**β = −.24;** ***SE*** **= .11;** ***t*(67) = −2.22;** ***p*** **= .029**
			rBA33	***R*** **= .50;** ***R***^**2**^ **= .25;** ***R***^**2**^_**adj**_ **= .18** ***F*(6, 67) = 3.68;** ***p*** **= .003;** ***SE*** **= 1.27**	β = −.03; *SE* = .11; *t*(67) = −.28; *p* = .776	β = −.13; *SE* = .11; *t*(67) = −1.19; *p* = .237	β = .17; *SE* = .12; *t*(67) = 1.40; *p* = .166	β = .14; *SE* = .12; *t*(67) = 1.18; *p* = .241	**β = .41;** ***SE*** **= .11;** ***t*(67) = 3.61;** ***p*** **< .001**	**β = −.22;** ***SE*** **= .11;** ***t*(67) = −2.02;** ***p*** **= .047**
		PCC	lBA23	***R*** **= .53;** ***R***^**2**^ **= .28;** ***R***^**2**^_**adj**_ **= .21** ***F*(6, 67) = 4.33;** ***p*** **= .001;** ***SE*** **= 1.86**	β = .19; *SE* = .11; *t*(67) = 1.77; *p* = .080	β = −.19; *SE* = .11; *t*(67) = −1.82; *p* = .072	β = .04; *SE* = .12; *t*(67) = .38; *p* = .703	β = .09; *SE* = .12; *t*(67) = .82; *p* = .412	**β = .45;** ***SE*** **= .11;** ***t*(67) = 4.08;** ***p*** **< .001**	β = −.21; *SE* = .11; *t*(67) = −1.98; *p* = .052
			rBA23	***R*** **= .55;** ***R***^**2**^ **= .30;** ***R***^**2**^_**adj**_ **= .24** ***F*(6, 67) = 4.82;** ***p*** **< .001;** ***SE*** **= 1.81**	β = .12; *SE* = .10; *t*(67) = 1.16; *p* = .250	β = −.21; *SE* = .10; *t*(67) = −1.98; *p* = .051	β = .07; *SE* = .11; *t*(67) = .58; *p* = .563	β = .10; *SE* = .11; *t*(67) = .90; *p* = .373	**β = .47;** ***SE*** **= .11;** ***t*(67) = 4.36;** ***p*** **< .001**	**β = −.25;** ***SE*** **= .11;** ***t*(67) = −2.39;** ***p*** **= .020**
			rBA30	***R*** **= .49;** ***R***^**2**^ **= .24;** ***R***^**2**^_**adj**_ **= .17** ***F*(6, 67) = 3.46;** ***p*** **= .005;** ***SE*** **= 2.96**	β = .05; *SE* = .11; *t*(67) = .42; *p* = .674	β = −.17; *SE* = .11; *t*(67) = −1.57; *p* = .120	β = .14; *SE* = .12; *t*(67) = 1.14; *p* = .260	β = .02; *SE* = .12; *t*(67) = .19; *p* = .850	**β = .41;** ***SE*** **= .11;** ***t*(67) = 3.66;** ***p*** **< .001**	β = −.16; *SE* = .11; *t*(67) = −1.40; *p* = .165
			rBA31	***R*** **= .60;** ***R***^**2**^ **= .35;** ***R***^**2**^_**adj**_ **= .30** ***F*(6, 67) = 6.16;** ***p*** **< .001;** ***SE*** **= 1.53**	β = .19; *SE* = .10; *t*(67) = 1.89; *p* = .062	**β = −.25;** ***SE*** **= .10;** ***t*(67) = −2.51;** ***p*** **= .014**	β = .02; *SE* = .11; *t*(67) = .19; *p* = .851	β = .14; *SE* = .11; *t*(67) = 1.32; *p* = .191	**β = .47;** ***SE*** **= .10;** ***t*(67) = 4.56;** ***p*** **< .001**	**β = −.32;** ***SE*** **= .10;** ***t*(67) = −3.13;** ***p*** **= .002**
		PFC	lBA11	***R*** **= .56;** ***R***^**2**^ **= .32;** ***R***^**2**^_**adj**_ **= .26** ***F*(6, 67) = 5.28;** ***p*** **< .001;** ***SE*** **= 3.07**	β = −.06; *SE* = .10; *t*(67) = −.45; *p* = .650	β = −.20; *SE* = .10; *t*(67) = −1.96; *p* = .055	β = .10; *SE* = .11; *t*(67) = .85; *p* = .397	β = .10; *SE* = .11; *t*(67) = .90; *p* = .373	**β = .48;** ***SE*** **= .11;** ***t*(67) = 4.47;** ***p*** **< .001**	**β = −.29;** ***SE*** **= .11;** ***t*(67) = −2.79;** ***p*** **= .007**
			rBA11	***R*** **= .48;** ***R***^**2**^ **= .23;** ***R***^**2**^_**adj**_ **= .16** ***F*(6, 67) = 3.38;** ***p*** **= .006;** ***SE*** **= 2.20**	β = −.01; *SE* = .11; *t*(67) = −.11; *p* = .911	β = −.09; *SE* = .11; *t*(67) = −.85; *p* = .396	β = .10; *SE* = .12; *t*(67) = .80; *p* = .428	β = .22; *SE* = .12; *t*(67) = 1.86; *p* = .067	**β = .42;** ***SE*** **= .11;** ***t*(67) = 3.70;** ***p*** **< .001**	β = −.18; *SE* = .11; *t*(67) = −1.65; *p* = .103
Ambiguous	P100	Limbic	rAMG	***R*** **= .50;** ***R***^**2**^ **= .26;** ***R***^**2**^_**adj**_ **= .19** ***F*(6, 67) = 3.84;** ***p*** **= .002;** ***SE*** **= 4.32**	β = .06; *SE* = .11; *t*(67) = .59; *p* = .560	β = −.12; *SE* = .11; *t*(67) = −1.16; *p* = .250	β = .16; *SE* = .12; *t*(67) = 1.34; *p* = .185	β = .12; *SE* = .12; *t*(67) = 1.05; *p* = .298	**β = .44;** ***SE*** **= .11;** ***t*(67) = 3.94;** ***p*** **< .001**	β = −.13; *SE* = .11; *t*(67) = −1.21; *p* = .230
			rAHj	***R*** **= .49;** ***R***^**2**^ **= .24;** ***R***^**2**^_**adj**_ **= .17** ***F*(6, 67) = 3.53;** ***p*** **= .004;** ***SE*** **= 4.12**	β = .05; *SE* = .11; *t*(67) = .43; *p* = .665	β = −.11; *SE* = .11; *t*(67) = −.98; *p* = .332	β = .16; *SE* = .12; *t*(67) = 1.39; *p* = .167	β = .14; *SE* = .12; *t*(67) = 1.14; *p* = .256	**β = .41;** ***SE*** **= .11;** ***t*(67) = 3.75;** ***p*** **< .001**	β = −.11; *SE* = .11; *t*(67) = −1.00; *p* = .322
			rHPC	***R*** **= .51;** ***R***^**2**^ **= .26;** ***R***^**2**^_**adj**_ **= .19** ***F*(6, 67) = 3.86;** ***p*** **= .002;** ***SE*** **= 4.10**	β = .06; *SE* = .11; *t*(67) = .53; *p* = .601	β = −.16; *SE* = .11; *t*(67) = −1.49; *p* = .142	β = .22; *SE* = .12; *t*(67) = 1.89; *p* = .063	β = .05; *SE* = .12; *t*(67) = .44; *p* = .663	**β = .40;** ***SE*** **= .11;** ***t*(67) = 3.58;** ***p*** **< .001**	β = −.10; *SE* = .11; *t*(67) = −.94; *p* = .352
		ACC	lBA32	***R*** **= .52;** ***R***^**2**^ **= .27;** ***R***^**2**^_**adj**_ **= .21** ***F*(6, 67) = 4.22** ***p*** **= .001;** ***SE*** **= 1.20**	β = −.05; *SE* = .11; *t*(67) = −.49; *p* = .622	β = −.18; *SE* = .11; *t*(67) = −1.73; *p* = .087	β = .15; *SE* = .12; *t*(67) = 1.34; *p* = .184	β = .14; *SE* = .12; *t*(67) = 1.24; *p* = .219	**β = .43;** ***SE*** **= .11;** ***t*(67) = 3.91;** ***p*** **< .001**	β = −.21; *SE* = .11; *t*(67) = −1.96; *p* = .053
			rBA32	***R*** **= .50;** ***R***^**2**^ **= .25;** ***R***^**2**^_**adj**_ **= .18** ***F*(6, 67) = 3.69;** ***p*** **= .003;** ***SE*** **= 1.01**	β = −.01; *SE* = .11; *t*(67) = −.12; *p* = .906	β = −.07; *SE* = .11; *t*(67) = −.66; *p* = .510	β = .18; *SE* = .12; *t*(67) = 1.54; *p* = .128	β = .18; *SE* = .12; *t*(67) = 1.52; *p* = .134	**β = .42;** ***SE*** **= .11;** ***t*(67) = 3.72;** ***p*** **< .001**	β = −.15; *SE* = .11; *t*(67) = −1.35; *p* = .181
		PCC	rBA23	***R*** **= .55;** ***R***^**2**^ **= .31;** ***R***^**2**^_**adj**_ **= .24** ***F*(6, 67) = 4.95;** ***p*** **< .001;** ***SE*** **= 2.13**	β = .20; *SE* = .10; *t*(67) = 1.90; *p* = .061	**β = −.21;** ***SE*** **= .10;** ***t*(67) = −2.04;** ***p*** **= .045**	β = .08; *SE* = .11; *t*(67) = .67; *p* = .508	β = .16; *SE* = .12; *t*(67) = 1.43; *p* = .157	**β = .44;** ***SE*** **= .11;** ***t*(67) = 4.08;** ***p*** **< .001**	**β = −.26;** ***SE*** **= .11;** ***t*(67) = −2.47;** ***p*** **= .016**
			rBA30	***R*** **= .48;** ***R***^**2**^ **= .23;** ***R***^**2**^_**adj**_ **= .16** ***F*(6, 67) = 3.40;** ***p*** **= .005;** ***SE*** **= 3.07**	β = .11; *SE* = .11; *t*(67) = 1.04; *p* = .302	β = −.17; *SE* = .11; *t*(67) = −1.59; *p* = .116	β = .16; *SE* = .12; *t*(67) = 1.35; *p* = .180	β = .04; *SE* = .12; *t*(67) = .35; *p* = .728	**β = .39;** ***SE*** **= .11;** ***t*(67) = 3.47;** ***p*** **= .001**	β = −.13; *SE* = .11; *t*(67) = −1.13; *p* = .262
		PFC	lBA11	***R*** **= .50;** ***R***^**2**^ **= .25;** ***R***^**2**^_**adj**_ **= .19** ***F*(6, 67) = 3.83;** ***p*** **= .002;** ***SE*** **= 3.41**	β = −.02; *SE* = .11; *t*(67) = −.19; *p* = .848	β = −.15; *SE* = .11; *t*(67) = −1.39; *p* = .169	β = .14; *SE* = .12; *t*(67) = 1.25; *p* = .214	β = .09; *SE* = .12; *t*(67) = .80; *p* = .427	**β = .41;** ***SE*** **= .11;** ***t*(67) = 3.71;** ***p*** **< .001**	**β = −.24;** ***SE*** **= .11;** ***t*(67) = −2.16;** ***p*** **= .034**
			rBA11	***R*** **= .49;** ***R***^**2**^ **= .23;** ***R***^**2**^_**adj**_ **= .16** ***F*(6, 67) = 3.32;** ***p*** **= .006;** ***SE*** **= 2.51**	β = .08; *SE* = .11; *t*(67) = .72; *p* = .474	β = −.09; *SE* = .11; *t*(67) = −.82; *p* = .413	β = .12; *SE* = .12; *t*(67) = 1.00; *p* = .321	β = .20; *SE* = .12; *t*(67) = 1.65; *p* = .102	**β = .41;** ***SE*** **= .11;** ***t*(67) = 3.62;** ***p*** **< .001**	β = −.15; *SE* = .11; *t*(67) = −1.34; *p* = .182
	N200	Limbic	rAMG	***R*** **= .49;** ***R***^**2**^ **= .24;** ***R***^**2**^_**adj**_ **= .17** ***F*(6, 67) = 3.53;** ***p*** **= .004;** ***SE*** **= 4.21**	β = .02; *SE* = .11; *t*(67) = .23; *p* = .820	β = −.10; *SE* = .11; *t*(67) = −.94; *p* = .352	β = .18; *SE* = .12; *t*(67) = 1.47; *p* = .146	β = .17; *SE* = .12; *t*(67) = 1.45; *p* = .152	**β = .41;** ***SE*** **= .11;** ***t*(67) = 3.64;** ***p*** **< .001**	β = −.15; *SE* = .11; *t*(67) = −1.37; *p* = .175
			rAHj	***R*** **= .47;** ***R***^**2**^ **= .22;** ***R***^**2**^_**adj**_ **= .15** ***F*(6, 67) = 3.20;** ***p*** **= .008;** ***SE*** **= 4.05**	β = .01; *SE* = .11; *t*(67) = .12; *p* = .908	β = −.08; *SE* = .11; *t*(67) = −.72; *p* = .471	β = .18; *SE* = .12; *t*(67) = 1.46; *p* = .149	β = .19; *SE* = .12; *t*(67) = 1.57; *p* = .120	**β = .39;** ***SE*** **= .11;** ***t*(67) = 3.44;** ***p*** **= .001**	β = −.12; *SE* = .11; *t*(67) = −1.06; *p* = .293
			rHPC	***R*** **= .49;** ***R***^**2**^ **= .24;** ***R***^**2**^_**adj**_ **= .18** ***F*(6, 67) = 3.60;** ***p*** **= .003;** ***SE*** **= 3.93**	β = .02; *SE* = .11; *t*(67) = .16; *p* = .876	β = −.13; *SE* = .11; *t*(67) = −1.22; *p* = .227	β = .22; *SE* = .12; *t*(67) = 1.89; *p* = .063	β = .09; *SE* = .12; *t*(67) = .73; *p* = .470	**β = .39;** ***SE*** **= .11;** ***t*(67) = 3.46;** ***p*** **= .001**	β = −.12; *SE* = .11; *t*(67) = −1.12; *p* = .265
	P250	Limbic	rAMG	***R*** **= .48;** ***R***^**2**^ **= .24;** ***R***^**2**^_**adj**_ **= .16** ***F*(6, 67) = 3.47;** ***p*** **= .005;** ***SE*** **= 4.24**	β = .04; *SE* = .11; *t*(67) = .38; *p* = .702	β = −.13; *SE* = .11; *t*(67) = −1.26; *p* = .211	β = .14; *SE* = .12; *t*(67) = 1.16; *p* = .250	β = .09; *SE* = .12; *t*(67) = .72; *p* = .471	**β = .43;** ***SE*** **= .11;** ***t*(67) = 3.79;** ***p*** **< .001**	β = −.14; *SE* = .11; *t*(67) = −1.29; *p* = .200
			rAHj	***R*** **= .47;** ***R***^**2**^ **= .22;** ***R***^**2**^_**adj**_ **= .15** ***F*(6, 67) = 3.22;** ***p*** **= .008;** ***SE*** **= 4.00**	β = .03; *SE* = .11; *t*(67) = .27; *p* = .789	β = −.11; *SE* = .11; *t*(67) = −1.08; *p* = .284	β = .14; *SE* = .12; *t*(67) = 1.17; *p* = .247	β = .10; *SE* = .12; *t*(67) = .84; *p* = .403	**β = .41;** ***SE*** **= .11;** ***t*(67) = 3.67;** ***p*** **< .001**	β = −.12; *SE* = .11; *t*(67) = −1.12; *p* = .264
			rHPC	***R*** **= .49;** ***R***^**2**^ **= .24;** ***R***^**2**^_**adj**_ **= .18** ***F*(6, 67) = 3.62;** ***p*** **= .003;** ***SE*** **= 4.03**	β = .02; *SE* = .11; *t*(67) = .21; *p* = .836	β = −.15; *SE* = .11; *t*(67) = −1.36; *p* = .177	β = .23; *SE* = .12; *t*(67) = 1.92; *p* = .060	β = .04; *SE* = .12; *t*(67) = .30; *p* = .762	**β = .38;** ***SE*** **= .11;** ***t*(67) = 3.39;** ***p*** **= .001**	β = −.11; *SE* = .11; *t*(67) = −1.01; *p* = .316
		ACC	lBA32	***R*** **= .54;** ***R***^**2**^ **= .29;** ***R***^**2**^_**adj**_ **= .23** ***F*(6, 67) = 4.61;** ***p*** **< .001;** ***SE*** **= 1.14**	β = −.10; *SE* = .10; *t*(67) = −.93; *p* = .354	β = −.18; *SE* = .11; *t*(67) = −1.74; *p* = .086	β = .14; *SE* = .11; *t*(67) = 1.21; *p* = .229	β = .12; *SE* = .11; *t*(67) = 1.06; *p* = .294	**β = .43;** ***SE*** **= .11;** ***t*(67) = 3.94;** ***p*** **< .001**	**β = −.27;** ***SE*** **= .11;** ***t*(67) = −2.52;** ***p*** **= .014**
		PCC	rBA23	***R*** **= .55;** ***R***^**2**^ **= .30;** ***R***^**2**^_**adj**_ **= .24** ***F*(6, 67) = 4.80;** ***p*** **< .001;** ***SE*** **= 2.04**	β = .16; *SE* = .10; *t*(67) = 1.59; *p* = .117	β = −.21; *SE* = .10; *t*(67) = −1.96; *p* = .053	β = .07; *SE* = .11; *t*(67) = .62; *p* = .535	β = .16; *SE* = .11; *t*(67) = 1.43; *p* = .157	**β = .44;** ***SE*** **= .11;** ***t*(67) = 4.05;** ***p*** **< .001**	**β = −.27;** ***SE*** **= .11;** ***t*(67) = −2.64;** ***p*** **= .010**
	LC1	Limbic	rAMG	***R*** **= .48;** ***R***^**2**^ **= .23;** ***R***^**2**^_**adj**_ **= .16** ***F*(6, 67) = 3.33;** ***p*** **= .006;** ***SE*** **= 4.20**	β = .07; *SE* = .11; *t*(67) = .68; *p* = .496	β = −.15; *SE* = .11; *t*(67) = −1.40; *p* = .166	β = .17; *SE* = .12; *t*(67) = 1.42; *p* = .158	β = .10; *SE* = .12; *t*(67) = .82; *p* = .413	**β = .40;** ***SE*** **= .11;** ***t*(67) = 3.52;** ***p*** **= .001**	β = −.12; *SE* = .11; *t*(67) = −1.11; *p* = .267
	LC2	Limbic	rAMG	***R*** **= .50;** ***R***^**2**^ **= .25;** ***R***^**2**^_**adj**_ **= .18** ***F*(6, 67) = 3.66;** ***p*** **= .003;** ***SE*** **= 4.12**	β = .03; *SE* = .11; *t*(67) = .30; *p* = .766	β = −.15; *SE* = .11; *t*(67) = −1.41; *p* = .164	β = .14; *SE* = .12; *t*(67) = 1.21; *p* = .231	β = .12; *SE* = .12; *t*(67) = 1.02; *p* = .312	**β = .44;** ***SE*** **= .11;** ***t*(67) = 3.86;** ***p*** **< .001**	β = −.15; *SE* = .11; *t*(67) = −1.33; *p* = .187
			rAHj	***R*** **= .48;** ***R***^**2**^ **= .23;** ***R***^**2**^_**adj**_ **= .16** ***F*(6, 67) = 3.32;** ***p*** **= .006;** ***SE*** **= 3.92**	β = .02; *SE* = .11; *t*(67) = .16; *p* = .872	β = −.13; *SE* = .11; *t*(67) = −1.21; *p* = .228	β = .16; *SE* = .12; *t*(67) = 1.29; *p* = .200	β = .13; *SE* = .12; *t*(67) = 1.11; *p* = .271	**β = .41;** ***SE*** **= .11;** ***t*(67) = 3.64;** ***p*** **< .001**	β = −.13; *SE* = .11; *t*(67) = −1.14; *p* = .256
			rHPC	***R*** **= .50;** ***R***^**2**^ **= .25;** ***R***^**2**^_**adj**_ **= .18** ***F*(6, 67) = 3.67;** ***p*** **= .003;** ***SE*** **= 3.85**	β = .02; *SE* = .11; *t*(67) = .16; *p* = .870	β = −.17; *SE* = .11; *t*(67) = −1.61; *p* = .113	β = .21; *SE* = .12; *t*(67) = 1.73; *p* = .088	β = .06; *SE* = .12; *t*(67) = .51; *p* = .614	**β = .40;** ***SE*** **= .11;** ***t*(67) = 3.54;** ***p*** **= .001**	β = −.12; *SE* = .11; *t*(67) = −1.08; *p* = .282
		PCC	rBA23	***R*** **= .55;** ***R***^**2**^ **= .30;** ***R***^**2**^_**adj**_ **= .24** ***F*(6, 67) = 4.83;** ***p*** **< .001;** ***SE*** **= 1.94**	β = .16; *SE* = .10; *t*(67) = 1.54; *p* = .127	β = −.19; *SE* = .10; *t*(67) = −1.85; *p* = .068	β = .07; *SE* = .11; *t*(67) = .62; *p* = .536	β = .18; *SE* = .11; *t*(67) = 1.55; *p* = .126	**β = .45;** ***SE*** **= .11;** ***t*(67) = 4.18;** ***p*** **< .001**	**β = −.26;** ***SE*** **= .11;** ***t*(67) = −2.44;** ***p*** **= .017**
			rBA30	***R*** **= .49;** ***R***^**2**^ **= .24;** ***R***^**2**^_**adj**_ **= .17** ***F*(6, 67) = 3.46;** ***p*** **= .005;** ***SE*** **= 2.86**	β = .05; *SE* = .11; *t*(67) = .48; *p* = .635	β = −.17; *SE* = .11; *t*(67) = −1.58; *p* = .118	β = .16; *SE* = .12; *t*(67) = 1.33; *p* = .187	β = .07; *SE* = .12; *t*(67) = .55; *p* = .581	**β = .41;** ***SE*** **= .11;** ***t*(67) = 3.60;** ***p*** **< .001**	β = −.15; *SE* = .11; *t*(67) = −1.33; *p* = .188

*Note.* AMG = amygdala; AHj = amygdala-hippocampus junction; HPC = hippocampus

*p* value < .05

**Fig. 3:**
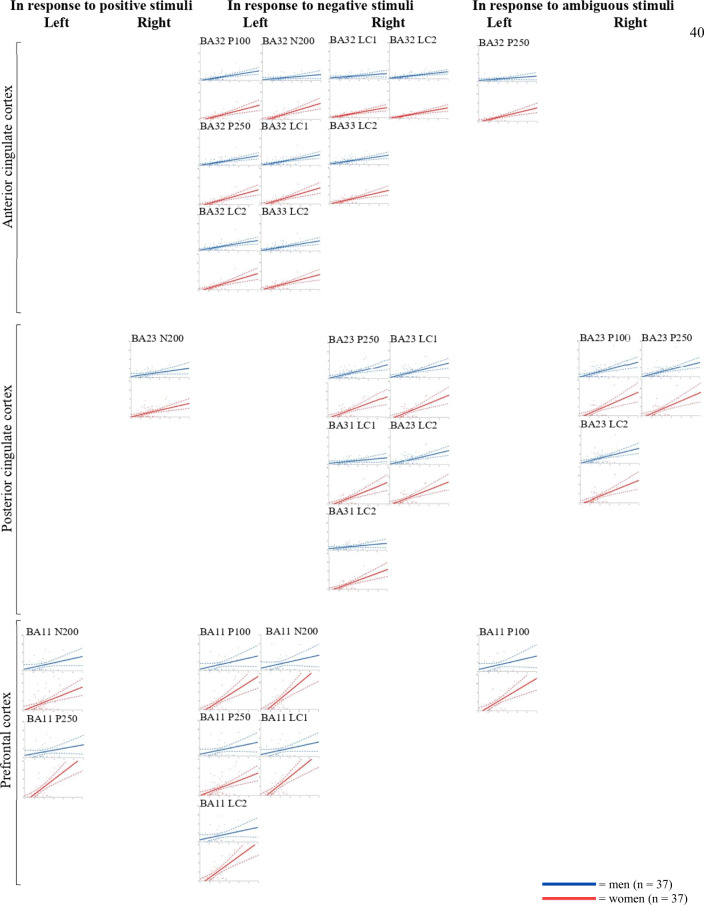
The associations in men and women between avoidance (*x*-axis) and the Brodmann areas (BA) intensity (*y*-axis) where the Sex × Avoidance interaction resulted as a significant predictor (.002 < *p* > .047) in the regression model (at P100, N200, P250, LC1, and LC2 components). In all the significant Sex × Avoidance interactions, the women showed more pronounced positive association compared with men. (*n* = 74)

As shown by the inclination of the regression lines in Fig. 3, the different avoidance/BA intensity associations in men versus women (represented by the significant Sex × Avoidance interaction in the regression models) showed a stronger positive association in women compared with men mostly for the late components of the cingulate and prefrontal cortices ROIs in response to negative condition.

## Discussion

The main finding of this study was that avoidance was positively associated with cortico-limbic brain intensity. Consistent with the hypotheses, this association presented a clear sex-related difference, for which the regression slopes in women were significantly more pronounced than those in men. As hypothesized, the findings of this study suggested that the avoidant attachment might be a sex-related adaptive strategy that involves different affective and cognitive processes in response to socioemotional distress. According to the attachment theory, the internal working models seem to be behavioral strategies that allow the regulation and maintenance of a minimum level of internal security, through the deactivation or hyperactivation of the attachment system (Mikulincer & Shaver, 2003).

The present findings suggested that in women, the avoidance strategy might be associated with an effort to regulate the hyperactivation of the attachment system induced by socioemotional cues, whereas the avoidance strategy in men appears to be associated with a more intense emotional suppression. One interpretation of this divergence between avoidant strategies in men and women is the general female tendency to be more receptive to emotional aspects in relational contexts (Proverbio et al., 2009). Several behavioral (Bachrach, Croon, & Bekker, 2015; Li & Fung, 2014) and neurobiological (George, Ketter, Parekh, Herscovitch, & Post, 1996; Piefke, Weiss, Markowitsch, & Fink, 2005; Proverbio et al., 2008) studies have supported the increased tendency among women to empathize and understand others and revealed the different cortico-limbic correlates in response to socioemotional stimuli between men and women.

In this study, the Sex × Avoidance interaction effect in regression analyses showed sex-related differences in the association between avoidance scores and brain intensity, particularly evident in response to the negative socioemotional stimuli, involving primarily the left and right anterior (BA32 and BA33) and posterior cingulate (BA23 and BA31) cortices in the late components and the left prefrontal cortex (BA11) in both early and late components. In response to the positive and ambiguous conditions, significantly different associations between men and women were less frequently observed, and primarily involved the right posterior cingulate cortex (BA23), the left anterior cingulate (BA32), and the left prefrontal cortices (BA11). These findings were also supported by the Sex × Condition interaction effect that as observed for the amplitudes of early ERP components, which confirmed the pivotal role of negative socioemotional cues for the activation of the attachment system, mostly in women. Consistently, recent studies (Proverbio et al., 2009; Stevens & Hamann, 2012) have demonstrated that women responded more strongly to negative emotional stimuli, particularly in the prefrontal brain areas, compared with men.

Moreover, the behavioral results of the present study revealed that men attributed fewer positive values to positive socioemotional stimuli than women did, which appears to suggest that men are less likely to express positive values on relational contexts (Tamres, Janicki, & Helgeson, 2002). As suggested by Buck and others (Buck, 1977, 1991; Chaplin, 2015; Levenson, Carstensen, & Gottman, 1994), this could indicate that men become internally aroused, but “keep in” their emotions through unknown regulatory mechanisms, whereas women express these emotions more freely. Consistent with this interpretation, in the present study, the ERP data showed a main effect of sex, in which women were observed to have shorter latencies and lower amplitudes within the early occipital and temporo-parietal components, and larger amplitudes in the late frontal component compared with those in men.

Coherently, in the regression analyses, sex was a significant predictor of the brain intensity, showing increased intensity in the cingulate and prefrontal cortices in women compared with that in men. Specifically, differences between men and women were found in the intensity levels of the left anterior cingulate (BA32), the right posterior cingulate (BA23 and BA31), and the left prefrontal cortex (BA11), in response to negative socioemotional stimuli, whereas differences were found only on the left prefrontal (BA11) in response to the positive condition and only on the right posterior cingulate cortex (BA23) in response to the ambiguous condition. These findings suggested that the women appeared to be more responsive to the socioemotional cues, primarily to the negative cues, compared with the responsiveness of men (Proverbio et al., 2009).

In contrast to our findings for avoidance, the anxiety dimension did not show any significant associations with any brain intensity, suggesting that anxiety may be less associated with the neurobiological pathways during socioemotional tasks (Ran & Zhang, 2018; Vrtička et al., 2008).

The ERP data showed that age was negatively associated with the latency of the P250 component in the frontal montage; however, this finding should be considered in light of the specific age range recruited for this study and which characterized the final sample (18–35-year-olds). This result could reflect the greater emotional involvement and the lowered inhibition in intimacy during relationships that occur with increasing of age, as reported in previous studies (Levenson et al., 1994; Radmacher & Azmitia, 2006).

Our study has some limitations. The scores of the attachment dimensions were assessed by a self-administered questionnaire, which could potentially favor social desirability bias and the acquiescent response bias. Due to the small age range of the participants (18–35 years), these findings cannot be generalized to populations with different ages.

In conclusion, the findings of the present study showed that women appear to be more emotionally involved during a socioemotional task. Avoidance was positively associated with the cingulate and prefrontal intensity levels, and these associations were more pronounced in women. These findings suggested that avoidance appears to represent two different socioemotional strategies, in which women appear to activate the avoidant strategy to modulate higher emotional involvement in relationships, whereas men appeared to adopt it with a more intense emotional suppression.

Whether these differences are inherited or whether they are linked to more complex sociocultural factors associated with the social learning of stereotypical sex roles remains unclear (Lungu, Potvin, Tikàsz, & Mendrek, 2015). How sociocultural factors modulate the associations between avoidance attachment and brain activation should be explored in the future.

From a clinical perspective, the findings of this study suggested that the avoidant attachment could require different levels of attention when treating men and women, with the treatment of avoidant women focused on the regulation of emotional hyperactivation.

In conclusion, based on the present results, future research that examines the neural correlates of avoidant attachment styles should consider sex-related differences.

## Supplementary Information

ESM 1 (DOCX 65 kb)

## References

[CR1] Althaus M, Groen Y, van der Schaft L, Minderaa RB, Tucha O, Mulder LJ, Wijers AA (2014). Sex differences in orienting to pictures with and without humans: Evidence from the cardiac evoked response (ECR) and the cortical long latency parietal positivity (LPP). PLOS ONE.

[CR2] Bachrach N, Croon MA, Bekker MH (2015). The role of sex, attachment and autonomy-connectedness in personality functioning. Personality and Mental Health.

[CR3] Bianchin M, Angrilli A (2012). Gender differences in emotional responses: A psychophysiological study. Physiology & Behavior.

[CR4] Bourisly AK, Shuaib A (2018). Sex differences in electrophysiology: P200 event-related potential evidence. Translational Neuroscience.

[CR5] Buchheim A, George C, Kächele H, Erk S, Walter H (2006). Measuring adult attachment representation in an fMRI environment: Concepts and assessment. Psychopathology.

[CR6] Buck R (1977). Nonverbal communication of affect in preschool children: Relationships with personality and skin conductance. Journal of Personality and Social Psychology.

[CR7] Buck, R. (1991). Temperament, social skills, and the communication of emotion. In D. G. Gilbert & J. J. Connolly (Eds.), *Personality, social skills, and psychopathology* (pp. 85–105). Springer. 10.1007/978-1-4899-0635-9_4

[CR8] Busonera A, Martini PS, Zavattini GC, Santona A (2014). Psychometric properties of an Italian version of the Experiences in Close Relationships–Revised (ECR-R) scale. Psychological Reports.

[CR9] Cecchini M, Aceto P, Altavilla D, Palumbo L, Lai C (2013). The role of the eyes in processing an intact face and its scrambled image: A dense array ERP and low-resolution electromagnetic tomography (sLORETA) study. Social Neuroscience.

[CR10] Cecchini M, Iannoni ME, Pandolfo AL, Aceto P, Lai C (2015). Attachment style dimensions are associated with brain activity in response to gaze interaction. Social Neuroscience.

[CR11] Chaplin TM (2015). Gender and emotion expression: A developmental contextual perspective. Emotion Review.

[CR12] Collignon O, Girard S, Gosselin F, Saint-Amour D, Lepore F, Lassonde M (2010). Women process multisensory emotion expressions more efficiently than men. Neuropsychologia.

[CR13] Collins NL, Feeney BC (2004). Working models of attachment shape perceptions of social support: Evidence from experimental and observational studies. Journal of Personality and Social Psychology.

[CR14] Dewall CN, Masten CL, Powell C, Combs D, Schurtz DR, Eisenberger NI (2012). Do neural responses to rejection depend on attachment style? An fMRI study. Social Cognitive and Affective Neuroscience.

[CR15] Fraley, R. C. (2012). Information on the Experiences in Close Relationships–Revised (ECR-R) Adult Attachment Questionnaire. Retrieved from http://labs.psychology.illinois.edu/~rcfraley/measures/ecrr.htm

[CR16] Fraley RC, Waller NG, Brennan KA (2000). An item response theory analysis of self-report measures of adult attachment. Journal of personality and social psychology.

[CR17] George MS, Ketter TA, Parekh PI, Herscovitch P, Post RM (1996). Gender differences in regional cerebral blood flow during transient self-induced sadness or happiness. Biological Psychiatry.

[CR18] Gillath O, Bunge SA, Shaver PR, Wendelken C, Mikulincer M (2005). Attachment-style differences in the ability to suppress negative thoughts: Exploring the neural correlates. NeuroImage.

[CR19] Guimond S, Chatard A, Martinot D, Crisp RJ, Redersdorff S (2006). Social comparison, self-stereotyping, and gender differences in self-construals. Journal of Personality and Social Psychology.

[CR20] Groen, Y., Wijers, A. A., Tucha, O., & Althaus, M. (2013). Are there sex differences in ERPs related to processing empathy-evoking pictures?. Neuropsychologia, 51(1), 142–155. 10.1016/j.neuropsychologia.2012.11.012 10.1016/j.neuropsychologia.2012.11.012 23174404

[CR21] Hampson E, van Anders SM, Mullin LI (2006). A female advantage in the recognition of emotional facial expressions: Test of an evolutionary hypothesis. Evolution and Human Behavior.

[CR22] Hazan C, Shaver P (1987). Romantic love conceptualized as an attachment process. Journal of Personality and Social Psychology.

[CR23] Krahe, C., Drabek, M. M., Paloyelis, Y., & Fotopoulou, A. (2016). Affective touch and attachment style modulate pain: A laser-evoked potentials study. *Philosophical Transactions of the Royal Society B: Biological Sciences, 371*(1708). 10.1098/rstb.2016.0009. 10.1098/rstb.2016.0009 PMC5062098 28080967

[CR24] Krahe C, Paloyelis Y, Condon H, Jenkinson PM, Williams SCR, Fotopoulou A (2015). Attachment style moderates partner presence effects on pain: A laser-evoked potentials study. Social Cognitive and Affective Neuroscience.

[CR25] Kret ME, De Gelder B (2012). A review on sex differences in processing emotional signals. Neuropsychologia.

[CR26] Lai C, Altavilla D, Mazza M, Scappaticci S, Tambelli R, Aceto P, Luciani M, Corvino S, Martinelli D, Alimonti F, Tonioni F (2017). Neural correlate of Internet use in patients undergoing psychological treatment for Internet addiction. Journal of Mental Health.

[CR27] Lai C, Altavilla D, Ronconi A, Aceto P (2016). Fear of missing out (FOMO) is associated with activation of the right middle temporal gyrus during inclusion social cue. Computers in Human Behavior.

[CR28] Lai C, Luciani M, Di Giorgio C, Fiorini R, Yaya G, Pellicano GR, Mazza M, Altavilla D, Aceto P (2018). Brain functional connectivity of meaning attribution in patients with psychosis: Preliminary electroencephalographic observations. Schizophrenia Research.

[CR29] Lai, C., Pellicano, G. R., Altavilla, D., Proietti, A., Lucarelli, G., Massaro, G., Luciani, M., & Aceto, P. (2018). Violence in video game produces a lower activation of limbic and temporal areas in response to social inclusion images. *Cognitive, Affective, & Behavioral Neuroscience*, 1–12. Advance online publication. 10.3758/s13415-018-00683-y 10.3758/s13415-018-00683-y 30565058

[CR30] Lai C, Pellicano GR, Ciacchella C, Guidobaldi L, Altavilla D, Cecchini M, Begotaraj E, Aceto P, Luciani M (2020). Neurophysiological correlates of emotional face perception consciousness. Neuropsychologia.

[CR31] Lancaster JL, Woldorff MG, Parsons LM, Liotti M, Freitas CS, Rainey L, Kochunov PV, Nickerson D, Mikiten SA, Fox PT (2000). Automated Talairach atlas labels for functional brain mapping. Human Brain Mapping.

[CR32] Lang, P. J., Bradley, M. M., & Cuthbert, B. N. (2008). *International Affective Picture System (IAPS): Affective ratings of pictures and instruction manual* (Technical Report A-8). Gainesville.

[CR33] Lee KH, Siegle GJ (2009). Common and distinct brain networks underlying explicit emotional evaluation: A meta-analytic study. Social Cognitive and Affective Neuroscience.

[CR34] Levenson RW, Carstensen LL, Gottman JM (1994). Influence of age and gender on affect, physiology, and their interrelations: A study of long-term marriages. Journal of Personality and Social Psychology.

[CR35] Li T, Fung HH (2014). How avoidant attachment influences subjective well-being: An investigation about the age and gender differences. Aging & Mental Health.

[CR36] Long M, Verbeke W, Ein-Dor T, Vrtička P (2020). A functional neuro-anatomical model of human attachment (NAMA): Insights from first-and second-person social neuroscience. Cortex.

[CR37] Luan J, Yao Z, Bai Y (2017). How social ties influence consumer: Evidence from event-related potentials. PLOS ONE.

[CR38] Luciani M, Cecchini M, Altavilla D, Palumbo L, Aceto P, Ruggeri G, Vecchio F, Lai C (2014). Neural correlate of the projection of mental states on the not-structured visual stimuli. Neuroscience Letters.

[CR39] Lungu O, Potvin S, Tikàsz A, Mendrek A (2015). Sex differences in effective fronto-limbic connectivity during negative emotion processing. Psychoneuroendocrinology.

[CR40] Main, M., Kaplan, N., & Cassidy, J. (1985). Security in infancy, childhood, and adulthood: A move to the level of representation. In I. Bretherton & E. Waters (Eds.), *Growing points in attachment theory and research: Monographs of the Society for Research in Child Development, 50* (1/2, Serial No. 209), 66–106. 10.2307/3333824

[CR41] Massaro G, Altavilla D, Aceto P, Pellicano GR, Lucarelli G, Luciani M, Lai C (2018). Neurophysiological correlates of collective trauma recall in 2009 L'Aquila earthquake survivors. Journal of Traumatic Stress.

[CR42] Mikulincer, M., & Shaver, P. R. (2003). The attachment behavioral system in adulthood: Activation, psychodynamics, and interpersonal processes. In M. P. Zanna (Ed.), *Advances in experimental social psychology* (Vol. 35, pp. 53–152). Elsevier. 10.1016/S0065-2601(03)01002-5

[CR43] Pascual-Marqui RD (2002). Standardized low-resolution brain electromagnetic tomography (sLORETA): Technical details. Methods and Findings in Experimental and Clinical Pharmacology.

[CR44] Picardi A, Vermigli P, Toni A, D’amico R, Bitetti D, Pasquini P (2002). Il questionario “Experiences in Close Relationships” (ECR) per la valutazione dell’attaccamento negli adulti: ampliamento delle evidenze di validità per la versione italiana [The “Experiences in Close Relationships” (ECR) questionnaire for the assessment of attachment in adults: Expansion of evidence of validity for the Italian version]. Giornale Italiano di Psicopatologia.

[CR45] Picton TW, Bentin S, Berg P, Donchin E, Hillyard SA, Johnson R, Miller GA, Ritter W, Ruchkin DS, Rugg MD, Taylor MJ (2000). Guidelines for using human event-related potentials to study cognition: Recording standards and publication criteria. Psychophysiology.

[CR46] Piefke M, Weiss PH, Markowitsch HJ, Fink GR (2005). Gender differences in the functional neuroanatomy of emotional episodic autobiographical memory. Human Brain Mapping.

[CR47] Proverbio AM, Adorni R, Zani A, Trestianu L (2009). Sex differences in the brain response to affective scenes with or without humans. Neuropsychologia.

[CR48] Proverbio, A. M., Zani, A., & Adorni, R. (2008). Neural markers of a greater female responsiveness to social stimuli. *BMC Neuroscience*, *9*(56). 10.1186/1471-2202-9-56 10.1186/1471-2202-9-56 PMC2453130 18590546

[CR49] Radmacher K, Azmitia M (2006). Are there gendered pathways to intimacy in early adolescents’ and emerging adults’ friendships?. Journal of Adolescent Research.

[CR50] Ran G, Zhang Q (2018). The neural correlates of attachment style during emotional processing: An activation likelihood estimation meta-analysis. Attachment & Human Development.

[CR51] Ratliff KA, Oishi S (2013). Gender differences in implicit self-esteem following a romantic partner’s success or failure. Journal of Personality and Social Psychology.

[CR52] Sander K, Frome Y, Scheich H (2007). FMRI activations of amygdala, cingulate cortex, and auditory cortex by infant laughing and crying. Human Brain Mapping.

[CR53] Schreiter ML, Chmielewski WX, Beste C (2018). How socioemotional setting modulates late-stage conflict resolution processes in the lateral prefrontal cortex. Cognitive, Affective, & Behavioral Neuroscience.

[CR54] Stevens JS, Hamann S (2012). Sex differences in brain activation to emotional stimuli: A meta-analysis of neuroimaging studies. Neuropsychologia.

[CR55] Strathearn L, Fonagy P, Amico J, Montague PR (2009). Adult attachment predicts maternal brain and oxytocin response to infant cues. Neuropsychopharmacology.

[CR56] Tamres LK, Janicki D, Helgeson VS (2002). Sex differences in coping behavior: A meta-analytic review and an examination of relative coping. Personality and Social Psychology Review.

[CR57] Tanner D, Morgan-Short K, Luck SJ (2015). How inappropriate high-pass filters can produce artifactual effects and incorrect conclusions in ERP studies of language and cognition. Psychophysiology.

[CR58] Vrtička P, Andersson F, Grandjean D, Sander D, Vuilleumier P (2008). Individual attachment style modulates human amygdala and striatum activation during social appraisal. PLOS ONE.

[CR59] Vrtička P, Bomdolfi G, Sander D, Vuilleumier P (2012). The neuronal substrates of social emotion perception and regulation are modulated by adult attachment style. Social Neuroscience.

[CR60] Vrtička, P., & Vuilleumier, P. (2012). Neuroscience of human social interactions and adult attachment style. *Frontiers in Human Neuroscience, 6*(212). 10.3389/fnhum.2012.00212 10.3389/fnhum.2012.00212 PMC3398354 22822396

[CR61] White LO, Wu J, Borelli JL, Rutherford HJ, David DH, Kim-Cohen J, Mayes LC, Crowley MJ (2012). Attachment dismissal predicts frontal slow-wave ERPs during rejection by unfamiliar peers. Emotion.

[CR62] Zayas V, Shoda Y, Mischel W, Osterhout L, Takahashi M (2009). Neural responses to partner rejection cues. Psychological Science.

